# RECQL4 requires PARP1 for recruitment to DNA damage, and PARG dePARylation facilitates its associated role in end joining

**DOI:** 10.1038/s12276-024-01383-z

**Published:** 2025-01-28

**Authors:** Mansoor Hussain, Prabhat Khadka, Komal Pekhale, Tomasz Kulikowicz, Samuel Gray, Alfred May, Deborah L. Croteau, Vilhelm A. Bohr

**Affiliations:** 1 Section on DNA Repair, Laboratory of Genetics and Genomics, National Institute on Aging, National Institutes of Health, Baltimore, MD USA; 2 Laboratory of Genetics and Genomics, National Institute on Aging, National Institutes of Health, Baltimore, MD USA; 3 Dept of ICMM, University of Copenhagen, Copenhagn, Denmark

**Keywords:** Mechanisms of disease, Double-strand DNA breaks

## Abstract

RecQ helicases, highly conserved proteins with pivotal roles in DNA replication, DNA repair and homologous recombination, are crucial for maintaining genomic integrity. Mutations in RECQL4 have been associated with various human diseases, including Rothmund–Thomson syndrome. RECQL4 is involved in regulating major DNA repair pathways, such as homologous recombination and nonhomologous end joining (NHEJ). RECQL4 has more prominent single-strand DNA annealing activity than helicase activity. Its ability to promote DNA damage repair and the precise role of its DNA annealing activity in DNA repair are unclear. Here we demonstrate that PARP1 interacts with RECQL4, increasing its single-stranded DNA strand annealing activity. PARP1 specifically promoted RECQL4 PARylation at both its N- and C-terminal regions, promoting RECQL4 recruitment to DNA double-strand breaks (DSBs). Inhibition or depletion of PARP1 significantly diminished RECQL4 recruitment and occupancy at specific DSB sites on chromosomes. After DNA damage, PARG dePARylated RECQL4 and stimulated its end-joining activity. RECQL4 actively displaced replication protein A from single-stranded DNA, promoting microhomology annealing in vitro. Furthermore, depletion of PARP1 or RECQL4 substantially impacted classical-NHEJ- and alternative-NHEJ-mediated DSB repair. Consequently, the combined activities of PARP1, PARG and RECQL4 modulate DNA repair.

## PARP1 enhances RECQL4 activity in DNA repair mechanisms

Cells have mechanisms to repair DNA damage, which is crucial for preventing diseases such as cancer. The authors wanted to understand how another protein, PARP1, affects the role of RECQL4 in DNA repair. The study involved laboratory experiments using human cells to see how RECQL4 and PARP1 interact. PARP1 helps recruit RECQL4 to sites of DNA damage and enhances its ability to repair DNA by promoting the strand annealing process. However, when RECQL4 is modified by PARP1 through PARylation, its repair activity is reduced. Another protein, PARG, can reverse this modification, restoring the function of RECQL4. The results suggest that the interaction between RECQL4 and PARP1 is important for efficient DNA repair. This understanding could lead to new strategies for treating diseases related to DNA repair defects, such as cancer.

This summary was initially drafted using artificial intelligence, then revised and fact-checked by the author.

## Introduction

DNA double-strand breaks (DSBs) are potentially lethal DNA lesions that commonly lead to chromosome rearrangements, genomic instability and/or tumorigenesis^1,2^. To protect against these consequences, cells utilize DNA repair pathways that remove and repair many different DNA lesions, including DSBs^3^. Five highly conserved human RecQ helicases, often called ‘guardians of the genome’, play important roles in repairing DNA lesions, ensuring chromosome stability and suppressing tumorigenesis^4^. These helicases are RECQL1, RECQL4, RECQL5, BLM and WRN^5–7^. Mutations in RECQL1, RECQL4, WRN and BLM are closely linked to the inherited autosomal recessive diseases RECON, Rothmund–Thomson, Werner and Bloom syndromes, respectively^4–6^. Mutations in RECQL4 are also associated with RAPADILINO and Baller–Gerold syndrome. These diseases are characterized by chromosomal instability, developmental abnormalities, cancer predisposition and premature aging^4,8^.

RECQL4 participates in multiple DNA repair pathways^9,10^, including nonhomologous end joining (NHEJ)-mediated and homologous recombination (HR)-mediated DSB repair (DSBR)^11,12^. RECQL4 contains the highly conserved RecQ helicase protein domain that encodes its 3′–5′ helicase activity (reviewed in ref.^13^). Its helicase activity is relatively weak compared with that of the other RecQ helicases, while it has robust single-strand DNA strand annealing activity^13–16^. Previously, it was thought that RECQL4 lacked helicase activity because of its pronounced annealing capability. However, research by Xu and colleagues revealed that RECQL4 does indeed exhibit helicase activity^16^. All human RecQ proteins possess ssDNA annealing activity. This annealing can be utilized in DNA replication, telomere maintenance, chromatin remodeling, DSBR and gene expression^17^. However, the exact role and biological relevance of RECQL4 ssDNA annealing are not understood.

We previously conducted comparative studies measuring the relative ssDNA annealing activities of the RecQ helicases. We identified that in the absence of ATP, RECQL4 and RECQL5 have the most efficient annealing activity^18^. To better understand the biological role of this activity, we screened for protein–protein interactors that might modulate functional activities among important DNA repair proteins in relevant pathways. We detected that poly(ADP‒ribose) polymerase 1 (PARP1) strongly stimulated the ssDNA annealing activity of RECQL4, indicating that this is a meaningful functional interaction.

Poly(ADP-ribosyl)ation (PARylation) is a post-translational modification that regulates DNA repair, chromatin remodeling, genetic stability, mitosis and cell death^19–21^. The most abundant enzyme that catalyzes protein PARylation is PARP1, the protein substrates of which include many DNA damage repair (DDR) proteins^22^. PARP1 itself is rapidly recruited to and activated at sites of DNA damage. Once activated, PARP1 consumes large amounts of NAD^+^ while undergoing autoPARylation and then PARylates target proteins, which helps recruit DNA repair proteins to DNA lesions to facilitate DNA repair^23–26^. PARP1 promotes HR and alternative (alt)-NHEJ-dependent DSBR, as well as base excision repair and single-strand break repair^27,28^. Poly(ADP‒ribose) glycohydrolase (PARG) is a key enzyme involved in the regulation of DNA repair, primarily through its role in reversing PARylation. PARG hydrolyzes PAR chains, effectively reversing PARP-mediated PARylation, a process referred to as dePARylation^29^. This activity is essential for regulating the dynamics of DNA repair processes. PARylated proteins are typically inactivated, and dePARylation by PARG restores their function, facilitating accurate DNA repair^30^.

PARG activity ensures that the DNA damage response is transient and reversible, preventing persistent PARylation, which could otherwise interfere with DNA repair processes and cellular functions. Defects in PARG or its dysregulation can lead to inefficient DNA repair and genomic instability and are implicated in carcinogenesis due to the accumulation of DNA lesions and mutations^31^. Thus, PARG plays a crucial role in maintaining genome integrity by regulating the repair of damaged DNA^32^.

PARP1 promotes the recruitment of DNA repair proteins to DSBs^27,33–38^. Consistently, compounds that inhibit PARP (PARPi) increase the amount of time required for other RecQ helicases, WRN and RECQL5, to colocalize to laser-induced DSBs^39^. So far, the functional interactions between PARP1 and RECQL4 or BLM are much less characterized than those between PARP1 and WRN or RECQL5 (ref.^39^). Here, we investigated whether the PARP1 protein and/or PARP1-dependent PARylation activity (PAR) influence the role and activity of RECQL4 in vitro and in vivo. We used biochemical assays to characterize how PARP1 affects the ssDNA annealing and helicase activities of RECQL4 and cellular assays to investigate how PARP1 recruits RECQL4 to DSBs. The results showed that PARP1 specifically stimulates the ssDNA annealing activity of RECQL4 and that PARP1 and PAR recruit RECQL4 to DSBs. The importance of these interactions was also evaluated in the context of cellular repair assays for NHEJ and alt-NHEJ or Theta-mediated end joining (TMEJ) DSBR, and these findings were then integrated into a model for the role of RECQL4 in DSBR.

## Materials and methods

### Cell lines, transfection and antibodies

HEK 293T, HeLa and U2OS cells were grown in Dulbecco’s modified Eagle medium (Gibco) supplemented with 10% fetal bovine serum and 1% penicillin–streptomycin at 37 °C in a 5% CO_2_ humidified incubator. The cells were purchased from American Type Culture Collection and tested for mycoplasma. For live-cell imaging experiments, 5 × 10^4^ cells were seeded in 2 cm glass-bottom plates. When the cells were 60–70% confluent, they were transfected with the indicated plasmids using Lipofectamine Plus (Invitrogen, Life Technologies) according to the manufacturer’s directions. The cells were used for microscopic analysis 24 h after transfection. For coimmunoprecipitation (co-IP) and biotin PAR pulldown assays, HEK293T or U2OS cells were plated at 60% confluence on 10 cm plates and transfected with 10 µg of plasmid per plate as suggested by the Lipofectamine Plus DNA transfection protocol. The cells were collected 48 h after transfection. The antibodies used were RECQL4 (made in house)^11,40^, BLM (NB100-214 Novus Biologicals), GFP (sc-8334, Santa Cruz), DNA ligase I (sc-20222, Santa Cruz), DNA ligase III (1F3, GeneTex), PARP1 (9542L, Cell Signaling), PAR (4336-BPC-100, Trevigen), XRCC1 (GTX23133, GeneTex), FLAG (F1804, Sigma), V5 (R960-CUS, Invitrogen) and Tubulin (sc-5286, Santa Cruz).

### Recombinant proteins

As previously described^18^, the recombinant RECQL4 protein was purified from the BL21 *Escherichia coli* system, and BLM was purified using a baculovirus/insect cell expression system. The active PARP1 enzyme was purchased from Active Motif (81737). PARG was purchased from BPS Bioscience (101726). PAR chains was purchased from Enzo (ALX-202-043).

### Coimmunoprecipitation and immunoblotting

HEK293T cells were transfected with various Flag-tagged RECQL4 constructs or with the indicated plasmids. Cell extracts were prepared with RIPA lysis buffer (Thermo Scientific Pierce) containing a protease inhibitor cocktail (Thermo Scientific Pierce) and benzonase (0.1 U/µl) with 1 µM Mg^2+^ was added to the lysates to eliminate DNA-mediated interactions. The lysates were clarified by centrifugation at 14,000*g* at 4 °C, and the cell extracts were obtained. Then, 100–200 µg of supernatants were incubated with either anti-IgG (control), anti-Flag Sepharose beads (Sigma-Aldrich) or an anti-PAR antibody (4336-BPC-100, Trevigen) for 2 h at 4 °C, followed by incubation with protein A/G magnetic beads (88802, Pierce) for 1 h at 4 °C. The beads were washed twice with 500 mM NaCl before being washed three times with IP buffer (1× PBS with 0.5% NP-40) and then boiled in 4× LDS dye (Invitrogen). The samples were loaded on SDS‒PAGE gels (Invitrogen) and then transferred to polyvinylidene difluoride (PVDF) membranes. The membranes were blocked with 5% nonfat milk in TBST for 45 min at room temperature, followed by incubation with primary antibody overnight at 4 °C. Horseradish peroxidase-conjugated secondary antibodies (Cell Signaling Technology) were then added to the membranes. The blots were developed via an enhanced chemiluminescence western blotting substrate (Perkin Elmer), and images were obtained via a Bio-Rad ChemiDoc system.

### Strand annealing assay

The DNA strand annealing activity of RECQL4 and BLM was measured using complementary oligonucleotides (the sequences are given in Table 1) to create duplex DNA. The top strand was labeled at the 5′-end using [γ-^32^P] ATP and T4 polynucleotide kinase. Annealing reactions (10 μl) were performed for 10 min at 37 °C in buffer (30 mM Tris–HCl pH 7.5, 50 mM KCl, 1 mM dithiothreitol (DTT), 5 mM MgCl_2_ and 100 µg/ml BSA) with the indicated amounts of RECQL4, BLM, PARP1 and PAR. The sequences of the duplex substrates are presented in Table 1. The reactions were stopped by the addition of stop buffer (10 mM EDTA, 10 mM Tris–Cl pH 8.0, 10% glycerol, 0.3% SDS, 0.05% bromophenol blue and 0.05% xylene cyanol). The reaction products were separated by electrophoresis on a 10% native polyacrylamide gel in 1× TBE running buffer at 200 V for 1 h. The gels were exposed to a PhosphorImager screen (GE Healthcare) and imaged with a Typhoon scanner (GE Healthcare). ImageLab version 6.0.1 (Bio-Rad) was used to analyze the phosphoimages and calculate the percentage of free versus annealed substrate in each reaction, which was normalized against the background. Assays were performed at least in triplicate and representative gels are shown.

**Table 1:** List of DNA sequences used in this study.

Structure	Substrate	Sequence (5′–3′)
Helicase assay	(a/b) 22/15 (fork-1)	T1 GGAATTCTACCAGTGCCTTGCTAGGACATCTTTGCCCA B1 CTAGACAGCTCCATGTAGCAAGGCACTGGTAGAATTC
Annealing assay	Blunt-end 80 base pairs	T3 GCTGATCAACCCTACATGTGTAGGTAACCCTAACCCTAACCCT AAGGACAACCCTAGTGAAGCTTGTAACCCTAGGAGCT B3 AGCTCCTAGGGTTACAAGCTTCACTAGGGTTGTCCTTAGGGTT AGGGTTAGGGTTACCTACACATGTAGGGTTGATCAGC
RPA assay	RP246 RP246c	GCTCTGATGCCGCATAGTTAAGCCAGCCCCGACACCCG CGGGTGTCGGGGCTGGCTTAACTATGCGGCATCAGAGC
MMEJ assay	NHEJ10-1 NHEJ10-2 NHEJ10-3 NHEJ10-4	GACTCACTGGTAGCTTAGACCAAAGAAAATCTGGTCAGCG GTCTAAGCTACCAGTGAGTC CAGTATCCTGTCACTCCAGTCAAAGAAAATCGCTGACCAG ACTGGAGTGACAGGATACTG

### Helicase assay

**Fig. 1:**
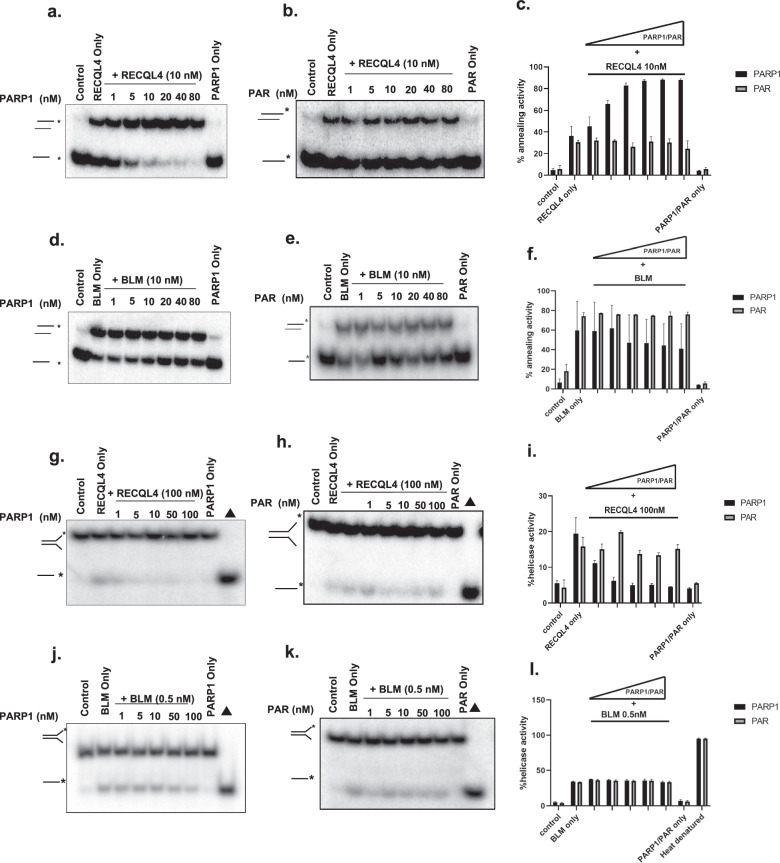
RECQL4-catalyzed strand annealing activity is stimulated by non-PARylated PARP1. **a**, **b**, The strand annealing activity of RECQL4 (10 nM) examined in the presence of increasing concentrations (0, 1, 5, 10, 20, 40 and 80 nM) of non-PARylated PARP1 (PARP1) (**a**) or PAR (**b**) with radiolabeled ssDNA 80 mer DNA and its complimentary single-stranded DNA. **c**, Graph showing the quantitative results of **a** and **b**. **d**, **e**, BLM (10 nM) strand annealing activity measured in the presence of increasing concentrations (0, 1, 5, 10, 20, 40 and 80 nM) of non-PARylated PARP1 (PARP1) (**d**) or PAR (**e**) with radiolabeled ssDNA 80 mer DNA and its complimentary single-stranded DNA. **f**, Graph showing the quantification results of **d** and **e. g**, **h**, Helicase activity of RECQL4 (100 nM) measured in the presence of increasing concentrations (1, 5, 10, 50 and 100 nM) of non-PARylated PARP1 (**g**) or PAR (**h**) with radiolabeled duplex fork DNA. **i**, A graph showing the quantitative results of **g** and **h**. **j**, **k**, BLM helicase activity (0.5 nM) was measured in the presence of increasing concentrations (1, 5, 10, 50 and 100 nM) of non-PARylated PARP1 (**j**) or PAR (**k**) with radiolabeled duplex fork DNA. **l**, Graph showing the quantification results of **j** and **k**. PARP1 and PAR alone have no helicase activity and ∆ represents the denatured substrate control. All experiments were repeated at least three times and the error bars represent the s.e.m.

Recombinant RECQL4 and BLM were purified as described previously^18^. PARP1 and PAR were obtained from Active Motif and Enzo. The helicase activities of RECQL4 and BLM with PARP1, PAR or PARylated PARP1 (at the concentrations shown in the figure 1) were measured using 0.5 nM radiolabeled fork duplexes (sequence given in Table 1) for 30 min at 37 °C in a volume of 10 µl of reaction buffer containing 30 mM Tris–HCl pH 7.4, 50 mM KCl, 5 mM MgCl_2_, 1 mM DTT, 100 µg/ml BSA, 10% glycerol, 5 mM ATP and complementary ssDNA substrates or forked duplex substrates, which have been described previously. The sequences of the forked duplex substrates are presented in Table 1. The reactions were stopped by the addition of stop buffer (10 mM EDTA, 10 mM Tris–Cl pH 8.0, 10% glycerol, 0.3% SDS, 0.05% bromophenol blue and 0.05% xylene cyanol). The reaction products were separated by electrophoresis on a 10% native polyacrylamide gel in 1× TBE running buffer at 200 V for 1 h. The gels were processed for the strand annealing assay.

### PAR overlay blot assay

The indicated amounts of recombinant proteins were vacuum blotted onto a nitrocellulose membrane using a slot-blot manifold^41^. The membrane was dried for 10 min at 50 °C, followed by incubation with PAR in TBS–Triton X100 (0.1%) overnight at 4 °C. Bound PAR was detected as described above using a 10H PAR antibody (Abcam, ab14459).

### Laser microirradiation and confocal microscopy

We used a Nikon Eclipse 2000E (Nikon) microscope equipped with a PerkinElmer Ultraview imaging system (PerkinElmer) and a MicroPoint Ablation system (Andor Technologies). The intensity of the laser (435 nM, 21%) was used to target the cells to generate DSBs, and all imaging aspects and analyses were performed using Volocity software 4.3.1 (Quorum Technologies, Inc.).

### RPA displacement and ssDNA annealing

First, 1 nM of ssDNA oligonucleotide RP246 (sequence given in Table 1) was incubated with replication protein A (RPA) at 37 °C for 5 min in reaction buffer (30 mM Tris–HCl pH 7.5, 5 mM ATP, 5 mM MgCl_2_, 1 mM DTT, 10% glycerol and 0.1 mg/ml BSA). Next, RECQL4 or BLM helicase was added, and the mixture was incubated for an additional 15 min at 37 °C. Finally, 0.5 nM of the 5′-^32^P-labeled complementary ssDNA oligonucleotide RP246c (sequence given in Table 1) was added, and the mixture was incubated for 10 min at 37 °C. The reactions (20 µl final volume) were terminated with 10 µl of 3× stop solution (30 mM Tris–HCl pH 8.0, 30 mM EDTA pH 8.0, 30% glycerol, 0.9% SDS, 40 nM cold RP246c oligo and 2% bromophenol blue) and separated on 10% native polyacrylamide gels. The reaction products were visualized using a Typhoon FLA 9500 phosphorimager (Cytiva) and analyzed with ImageQuant TL 8.2 software (Cytiva).

### Microhomology annealing assay

A 10 bp DNA microhomology substrate was constructed using the following oligos: NHEJ10-1, NHEJ10-2, NHEJ10-3 and NHEJ10-4 (the sequences are given in Table 1). NHEJ10-3 was labeled with 5′-FAM, and the corresponding oligonucleotides were annealed to form the duplexes of NHEJ10-1/2 and FAM-NHEJ-3/4. A mixture of 1 nM FAM-NHEJ-3/4 and 2 nM NHEJ10-1/2 was incubated with RECQL4 in the presence or absence of PARP1, PARG, NAD^+^ or PARylated PARP1 for 5 min at 37 °C in reaction buffer (30 mM Tris–HCl pH 7.5, 5 mM MgCl_2_, 1 mM DTT, 10% glycerol and 0.1 mg/ml BSA). The reactions (20 µl final volume) were terminated with 10 µl of 3× stop solution (30 mM Tris–HCl pH 8.0, 30 mM EDTA pH 8.0, 30% glycerol, 0.9% SDS and 2% bromophenol blue) and separated on 8% native polyacrylamide gels. The reaction products were visualized using a Typhoon FLA 9500 phosphorimager (Cytiva) and analyzed with ImageQuant TL 8.2 software (Cytiva).

### Chromatin extraction

Chromatin was extracted using a commercial kit (Abcam, ab117152). Briefly, 10^7^ cells were pelleted, washed with PBS, added to a working lysis buffer and transferred to a 1.5 ml vial on ice for 10 min. The samples were vortexed vigorously for 10 s and centrifuged at 5,000 rpm for 5 min. The supernatants were carefully removed and mixed with 500 μl (1 × 10^6^ cells/50 μl) of working extraction buffer on ice for 10 min and vortexed occasionally. The samples were sonicated 2× for 20 s to increase the degree of chromatin extraction. The samples were cooled on ice between sonication pulses for 30 s and centrifuged at 12,000 rpm at 4 °C for 10 min. The supernatants were transferred to a new vial, and chromatin buffer was added at a ratio of 1:1. The proteins were then separated by SDS‒PAGE. Detection via western blotting was performed with the appropriate antibodies.

### ChIP

For the chromatin immunoprecipitation (ChIP)‒qPCR experiments, monoclonal U2OS AsiSi cells were plated on 15 cm culture dishes. At 12 h postplating, 4-hydroxytamoxifen (4-OHT) was added for the cellular translocation of the AsiSi endonuclease from the cytoplasm to the nucleus. Then, at 4 h postinduction the cells were collected for ChIP experiments. We strictly followed the manufacturer’s protocol from the SimpleChIP Kit (Cell Signaling, CST 9003S). The following antibodies were used: anti-RECQL4 (rabbit mAb (Novus, NBP2-47310)) and normal rabbit IgG (Sigma,12-370). For the immunoprecipitation (IP) reactions, the samples were incubated with appropriate antibodies for 2 h at 4 °C with rotation. The DNA products were purified via a simple ChIP kit and quantified by qPCR using the DyNAmo HS SYBR Green qPCR kit (F-410L, Thermo Fisher Scientific) on the iQ5 and CFX Connect Real-time PCR Detection System (Bio-Rad). The following qPCR primers were used:

Primer name distance from the AsiSI oligo sequence (5′ → 3′)

No DSB FWD ATTGGGTATCTGCGTCTAGTGAGG

REV GACTCAATTACATCCCTGCAGCT

DSB1 180 bp FWD TGTGGACTCAGGGAACTC

REV CAGTCGCATACATCCGAT DSB1

DSB1 335 bp FWD GAATCGGATGTATGCGACTGATC

REV TTCCAAAGTTATTCCAACCCGAT

DSB1 1,618 bp FWD TGAGGAGGTGACATTAGAACTCAGA

REV AGGACTCACTTACACGGCCTTT.

#### In vivo DSBR assays

EJ2 and EJ5 U2OS cells were used for the in vivo DSBR assays as described previously^42,43^. The cells were transfected with the indicated small interfering (si)Control, siRECQL4 and siBLM constructs (GE Healthcare) by jetPRIME for siRNA (INTERFERin): siRECQL4 sequence, GUAAACAGCUCCUGAAAGA; siBLM sequence, CCGAGAAATCTCTTACCTCAA. The negative control siRNA was purchased from Qiagen (1027310). In vivo NHEJ assays were performed with the EJ2 and EJ5 reporting systems. To measure the efficiency of alt-NHEJ and NHEJ in EJ2 and EJ5 cells, DSBs were induced in the EJ2 and EJ5 reporter cassettes via the transfection of 2.5 µg of the I-SceI plasmid into 1 × 10^6^ cells using the Amaxa Cell Line Nucleofector Kit V (Lonza) following the manufacturer’s protocol. For normalization, the cells were transfected with 25 ng of pDsRed-Express-C1 along with the I-SceI plasmid. For the knockdown experiments, 24 h post-siRNA transfection, 1 × 10^5^ cells were transfected with I-SceI and pDsRed-Express-C1. At 4 days after I-SceI transfection, the cells were collected and analyzed by flow cytometry for the expression of GFP and DsRed. The data were collected on a BD Accuri C6 flow cytometer, and 50,000 cells were scored. To inhibit PARP1 activity, EJ2 and EJ5 cells transfected with plasmids expressing I-SceI and DsRed were treated with 5 µM olaparib for 4 days.

### Proximity ligation assay

The proximity ligation assay (PLA) was performed according to the manufacturer’s protocol (Duolink, Sigma-Aldrich). The primary antibodies used in this study were anti-Flag (F3165, Sigma) and anti-PARP1 (39061, Active Motif) antibodies. The samples were mounted with Duolink In Situ Mounting Medium with 4,6-diamidino-2-phenylindole (DAPI) for 15 min at room temperature and analyzed using a Zeiss Observer Z1. The resulting images and PLAs were then measured using CellProfiler 4.2.4 software.

### Clonogenic assay

DLD1 wild-type (WT) (1 × 10^5^) cells were transfected with control or siBRCA2 siRNAs with or without RECQL4 (50 nM) and plated (1000 cells) in 6-well tissue culture dishes at 72 h post-transfection. Drugs were added to the medium 4 h after cell seeding, and the cells were allowed to grow colonies in the presence of drugs for 15 days. A colony was defined as a cluster of at least 50 cells. Colonies were washed twice with 1× PBS, fixed with 100% methanol and stained with crystal violet staining solution (25% methanol + 0.5% crystal violet). The percentage of survival upon drug treatment was determined by normalizing the total number of colonies formed to the plating efficiency under individual conditions. Graphs were generated using GraphPad Prism, version 5.03.

### MMEJ assay in nuclear extracts

The microhomology-mediated end joining (MMEJ) assay was performed as described by Vekariya et al.^30^, with modifications. Briefly, asynchronously growing U2OS cells 90% confluent and transiently expressing FLAG–RECQL4, GFP–PARG or V5–PARP1 in 60 mm plates were irradiated with X-rays (10 Gy). After 60 min of incubation, the irradiated and control cells were harvested for the preparation of nuclear extracts. Next, 100 ng of I-SceI-digested pBabe-hygro-EGFP-MMEJ, the repair substrate, was mixed with 100 μl of nuclear extracts for 30 min with gentle shaking at 30 °C, followed by incubation for 15 h at 16 °C. A total of 10 μl of the mixture was transfected into XL-10-gold ultracompetent *E.* *coli* (Agilent Technologies) following the manufacturer’s protocol. The colonies on each agar plate were counted and plotted using GraphPad Prism 5.03. Three biological experiments were performed, and the ±s.d. values are shown on the graph.

## Results

### Non-PARylated PARP1 stimulates the strand annealing activity of RECQL4

RECQL4 is involved in multiple DNA repair pathways and its helicase function is well documented. However, its annealing activity has not been well explored. Our previous study revealed that among the RecQ helicases, RECQL4 has the most robust ssDNA annealing activity^18^. As the biochemical activities of RecQ helicases are regulated by interacting proteins^18^, we explored whether PARP1 affects the ssDNA annealing activities of RECQL4 and BLM. These two helicases are recruited early in response to DNA damage and participate in multiple DNA repair pathways, and comparisons between these two helicases are of particular interest, as reported in previous studies including our own^6,18^. These helicases were assayed for ssDNA annealing activity in the presence of increasing amounts of non-PARylated PARP1 (PARP1) (1–80 nM), PAR chains or PARylated PARP1. The results revealed that non-PARylated PARP1, but not PAR/PARylated PARP1, stimulates the ssDNA annealing activity of RECQL4 approximately twofold in a dose-dependent manner (Fig. 1a–c and Supplementary Fig. 1a). In contrast, neither non-PARylated PARP1 nor PAR substantially influenced the efficiency of the ssDNA annealing activity of BLM (Fig. 1d–f), suggesting that it is specific to RECQL4. These data are summarized in Table 2.

**Table 2:** Summary of the effects of PARP1 on human RecQ proteins in the presence of RecQ-interacting proteins.

Interacting partners	Strand annealing			Helicase activity		
	RECQL4	BLM	RECQ5	RECQL4	BLM	RECQ5
PARP1	Greater stimulation (this study)	Minimal inhibition (this study)	Inhibited^39^	Greater inhibition (this study)	NA (this study)	Greater inhibition^39^
PAR	NA (this study)	NA (this study)	Greater stimulation^39^	NA (this study)	NA (this study)	Inhibited^39^

RECQL4 and BLM DNA strand annealing and helicase activity were assessed in the presence of PARP1 and PAR. *NA* not affected.

### RECQL4-catalyzed helicase activity is inhibited by PARP1

Previously, we showed that PARP1 and PAR inhibit the helicase activity of RECQL5 and WRN^39^. Here, we examined the effects of PARP1 and PAR on RECQL4 and BLM helicase activities using a forked-heteroduplex DNA substrate. It has been previously reported that RECQL4 exhibits weak helicase activity related to its strong intrinsic strand annealing activity in vitro^13,15,40^. Our results revealed that the helicase activity of RECQL4 was inhibited in a dose-dependent manner by PARP1 but was unaffected by the presence of PAR (Fig. 1g–i), whereas the helicase activity of BLM was unaffected by the presence of either PAR or PARP1 (Fig. 1j–l). These data are summarized in Table 2. PARP1 possesses unique DNA binding motifs^44^. Surprisingly, the introduction of PARP1 did not impede BLM helicase function; instead, it selectively hindered only the helicase activity of RECQL4. This observation indicates that the ability of PARP1 to inhibit RECQL4 helicase activity may not be attributable solely to the DNA-binding capacity of PARP1.

### PARP1-mediated PARylation is required for the early recruitment of RECQL4 to DSBs

**Fig. 2:**
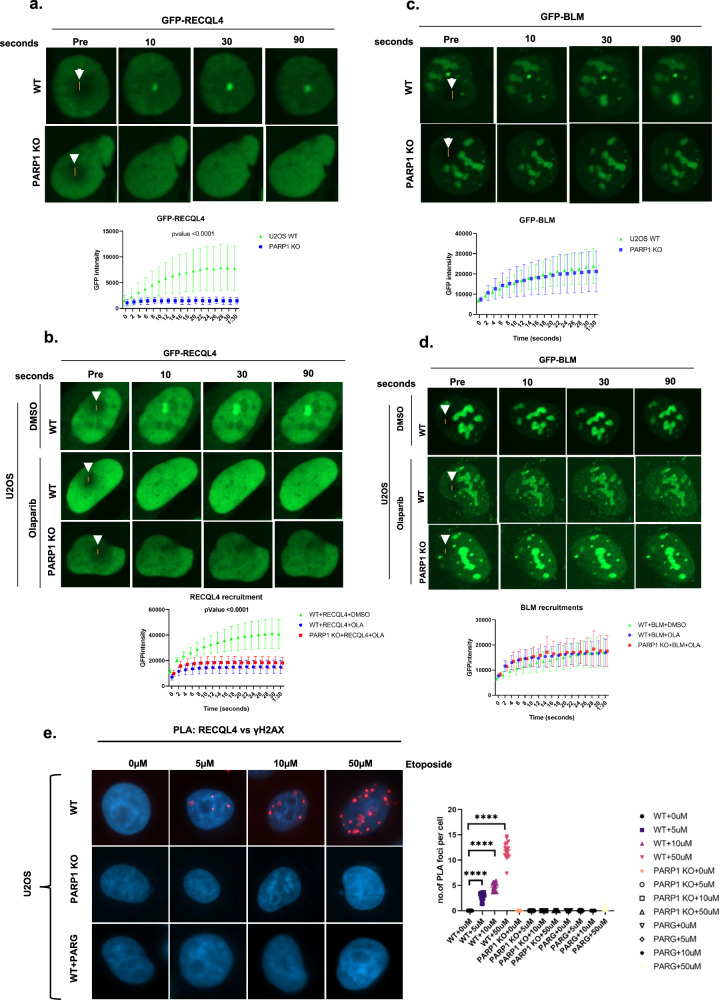
PARP1 is required for the early recruitment of RECQL4 to sites of DNA damage. **a**, RECQL4 recruitment to laser-induced DNA damage. GFP-tagged RECQL4 was transiently transfected into U2OS cells (WT or PARP1 KO) for 24 h. The cells were targeted with a 21% laser to induce DSBs. The cells were imaged at the indicated time points to observe the recruitment of the proteins to the damaged DNA. The recruitment kinetics of cells from three independent experiments were quantified for GFP–RECQL4. **b**, RECQL4, showing the same type of cells (U2OS) that were pretreated for 3 h with 5 µM olaparib and then targeted with the 21% laser to induce DSBs. **c**, GFP-tagged BLM was transiently transfected into U2OS cells (WT or PARP1 KO) for 24 h. The cells were targeted with a 21% laser to induce DSBs. The cells were imaged at the indicated time points to observe the recruitment of the proteins to the damaged DNA. The recruitment kinetics of cells from three independent experiments were quantified for GFP–BLM. **d**, GFP–BLM image showing the same type of cells (U2OS) that were pretreated for 3 h with 5 µM olaparib and then targeted with the 21% laser to induce DSBs. In **a**–**d**, the lower graphics represent the relative signal intensities at the laser line calculated using volocity software and plotted versus time. The white arrow indicates the laser striking area. The error bars represent the s.e.m. Two-way analysis of variance (ANOVA) was performed to assess significant differences (*P < 0.05, **P < 0.01 and ***P < 0.001). **e**, Proportional increase in RECQL4–γH2AX foci in response to etoposide dosage. U2OS cells, including WT, PARP1 KO and PARG-expressing cells, were treated with either dimethylsulfoxide (DMSO) or specified concentrations of etoposide for 2 h. PLA staining was subsequently performed with anti-RECQL4 and anti-γH2AX antibodies. The graph shows the number of PLA foci per cell under various treatment conditions. Statistical analysis was conducted using two-way ANOVA to evaluate significant differences (*P < 0.05, **P < 0.01 and ****P < 0.0001).

PARP1 directly recognizes DNA DSBs or single-strand breaks and promotes the rapid recruitment of DDR proteins to damaged DNA in a PAR-dependent manner^36,45^. Previously, we demonstrated that RECQL4 is recruited to DSB sites as early as 2 s after DNA damage and is independent of the DDR proteins ATM and DNA-PKcs^14^. Since PARP1 alters RECQL4 function (Fig. 1), determining whether PARP1 plays a role in the recruitment of RECQL4 to DNA damage sites is crucial. To investigate this role, we used U2OS WT or PARP1 knockout (KO) cells (Supplementary Fig. 2) to investigate the role of PARP1 in RECQL4 recruitment. Equal amounts of GFP-tagged RECQL4/BLM were expressed in U2OS WT/PARP1 KO cells (Supplementary Fig. 3), and RECQL4 recruitment to DSBs was examined using 21% laser microirradiation, which is known to induce DSBs^14,46^. As depicted in Fig. 2a, GFP–RECQL4 is not recruited to DSBs in PARP1 KO cells but is recruited in cells expressing GFP–RECQL4 and PARP1. Olaparib, a PARP1 and PARP2 inhibitor (PARPi), also severely inhibited the recruitment of GFP–RECQL4 to DSBs (Fig. 2b). Similar results were obtained in PARP1 KO cells and in PARPi inhibitor-treated HeLa cells (Supplementary Fig. 4a,b); thus, the PARP1-dependent early recruitment of RECQL4 to DSB sites is not cell type specific. No recruitment was observed in U2OS cells expressing GFP alone (Supplementary Fig. 4c).

PARP1 is responsible for 90% of the synthesized PAR chains in cells in response to DNA damage, and these chains are rapidly hydrolyzed by the exo- and endoglycosidase activity of PARG^47^. Thus, we tested how PARG might alter the recruitment of RECQL4 by expressing PARG and CFP–RECQL4 in U2OS WT cells, subjecting the cells to laser microirradiation, and monitoring CFP–RECQL4 localization. While the expression of PARG completely suppressed stable CFP–RECQL4 recruitment, treatment with ATRi or DNA PKci (Supplementary Fig. 4d) did not. Thus, RECQL4 recruitment to DSBs requires the PARP-mediated synthesis of PAR chains.

RECQL4 interacts with BLM and stimulates BLM helicase activity on DNA fork substrates in vitro^48^. This functional interaction led us to investigate whether BLM recruitment was also affected by PARP1. However, PARP1 KO or pretreatment of cells with a PARPi did not affect the recruitment of GFP–BLM to DSBs (Fig. 2c, d). Similar results were obtained in PARP1 KO cells and PARPi inhibitor-treated HeLa cells (Supplementary Fig. 4e,f).

Since laser-induced DNA damage can lead to various types of DNA damage, we also used the topoisomerase II poison etoposide to induce more specific DNA DSBs. We performed PLAs using RECQL4 and γH2AX antibodies to examine RECQL4 recruitment to DNA damage sites after treating cells with different concentrations of etoposide. As shown in Fig. 2e, U2OS WT cells exhibited positive PLA foci in a dose-dependent manner. In contrast, PARP1 KO and PARG-expressing WT cells (Supplementary Fig. 5a) lacked PLA foci (Fig. 2e). Similar results were observed in the chromatin fractionation assays. Etoposide treatment of U2OS WT cells increased RECQL4 binding to chromatin in a dose-dependent manner, whereas this effect was not observed in PARP1 KO or PARG-expressing cells (Supplementary Fig. 5b).

Recruitment of RECQL5 and WRN is also delayed in PARP1 KO cells and WT cells treated with PARPis^39^. Thus, the impact of PARP1 depletion on recruitment to DSBs differs for RECQL4 compared with WRN, BLM and RECQL5. For RECQL4, recruitment is completely diminished in the absence of PARP1, whereas for WRN, BLM and RECQL5, the kinetics of recruitment are slower in the absence of PARP1. These results are summarized in Table 3.

**Table 3:** Summary of the effects of PARP1 on the recruitment of RECQL4 and BLM at DSB sites.

Cell line or treatment	Recruitment at DSB site	
	RECQL4	BLM
PARP1 KO cell line	No recruitment compared with WT cells	Delayed recruitment relative to WT cells
+ PARP1 inhibitor (olaparib)	No recruitment compared with WT cells	Delayed recruitment relative to WT cells

### RECQL4 interacts with PARP1

**Fig. 3:**
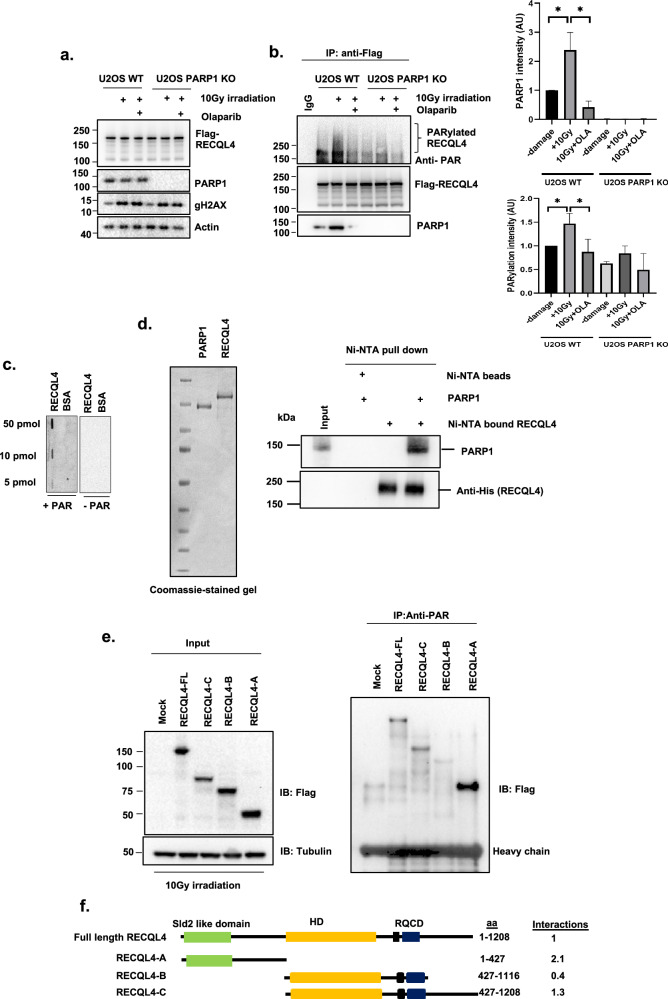
RECQL4 interacts with PARP1 and PAR. **a**, U2OS WT cells were transfected with the indicated plasmids and treated with or without 5 µM olaparib for 3 h before 10 Gy of IR DNA damage. Protein inputs were assessed by western blotting with the indicated antibodies. Actin served as the endogenous loading control. **b**, Whole-cell extracts from U2OS cells were immunoprecipitated with anti-Flag beads and immunoblotted with anti-PAR, anti-PARP1 and anti-Flag antibodies (left). Densitometric analysis was performed to measure the extent of the RECQL4‒PARP1 interaction and RECQL4 PARylation (right). *N* = 3, a Student’s *t*-test (two-sided) was performed to assess statistical significance (*P < 0.05, **P < 0.01 and ***P < 0.001) **c**, Slot‒blot assays were used to analyze the binding of purified RECQL4 proteins (50, 10 and 5 pmol) to PAR (how much PAR was loaded on a blot) that were dot blotted onto a nitrocellulose membrane. BSA (50, 10 and 5 pmol) was used as a negative control. **d**, The interaction between RECQL4 and PARP1 was investigated. Purified recombinant RECQL4 bound to Ni-NTA beads and soluble PARP1 were detected via Coomassie-stained gel (left). The interaction between bound RECQL4 and soluble PARP1 was analyzed via western blotting with anti-His (for RECQL4) and anti-PARP1 antibodies (right). **e**, PAR pulldown assays with RECQL4 fragments. After 10 Gy of IR damage, cell lysates were prepared from cells overexpressing either control Flag-tagged RECQL4 or various Flag-tagged RECQL4 domains. These lysates were incubated with an anti-PAR antibody to pull down PARylated proteins. The immunoprecipitants were then probed with an anti-Flag antibody. **f**, An illustration of Flag-tagged RECQL4 full-length RECQL4 and its fragments used for the binding assay. The values represent the degree of PARylation, which was determined through densitometry analysis of the pulled-down RECQL4 fragments relative to their initial input. This method quantifies PARylation on RECQL4.

PARP1 recognizes DNA breaks and PARP1 activity is involved in the recruitment of multiple DNA repair proteins^25^. Our in vitro assays revealed that non-PARylated PARP1 enhances RECQL4 annealing. To further investigate the interaction between RECQL4 and PARP1, Flag-tagged RECQL4 was expressed in U2OS WT/PARP1 KO cells before and after gamma irradiation (IR) and in the presence or absence of olaparib. The interaction between PARP1 and RECQL4 and RECQL4 PARylation were analyzed by immunoprecipitating Flag-tagged RECQL4 and probing for PARP1 and anti-PAR antibodies. IR treatment induced γH2AX but did not change the protein levels of RECQL4 or PARP1 (Fig. 3a). The immunoprecipitants were treated with benzonase to remove DNA and were then analyzed via antibodies against the Flag-tag and endogenous PARP1. The results revealed a basal association between RECQL4 and PARP1 without DNA damage exposure. Interestingly, DNA damage significantly enhanced the association of RECQL4 with PARP1, and treatment with olaparib, a PARP1 inhibitor, reduced this interaction (Fig. 3b). These findings indicate that PARylation plays a crucial role in the RECQL4‒PARP1 interaction. As co-IP was performed in the presence of a nuclease or benzonase, the interaction is not bridged by DNA. These findings are in agreement with a previous report showing that RECQL4 and PARP1 interact in human cells^49^.

To further investigate whether PARP1 targets RECQL4 for PARylation, benzonase-treated immunoprecipitates were probed with an anti-PAR antibody. As shown in Fig. 3b, DNA damage increased the number of PAR streaks on RECQL4, whereas PARP1KO cell/olaparib-treated cells did not stimulate RECQL4 PARylation. These findings indicate a crucial role of PARP1 in RECQL4 PARylation in response to DNA damage.

In another experiment (Fig. 3b), we used a PAR antibody to detect RECQL4 PARylation. To assay the PAR-binding ability of RECQL4, slot blots^41^ were used to directly demonstrate that RECQL4 binds to PAR in vitro (Fig. 3c). In addition, in vitro pulldown assays using purified recombinant His–RECQL4 or Ni–NTA alone further corroborated the RECQL4‒PARP1 interaction (Fig. 3d). To analyze the domain in RECQL4 that undergoes PARylation, protein extracts of HEK293T cells expressing Flag-tagged RECQL4 (full-length or three truncated Flag-tagged variants) were used (Fig. 3e, left) after 10 Gy of gamma IR. The full-length Flag-tagged RECQL4, the N-terminal region and the C-terminal region of the RECQL4 variant were pulled down with anti-PAR antibodies (Fig. 3e, right). The PARylated domains on RECQL4 are summarized in Fig. 3f. These findings suggest that there are PARylation sites within both the N- and C-terminal regions of RECQL4.

### Identification of interaction domains between PARP1 and RECQL4

**Fig. 4:**
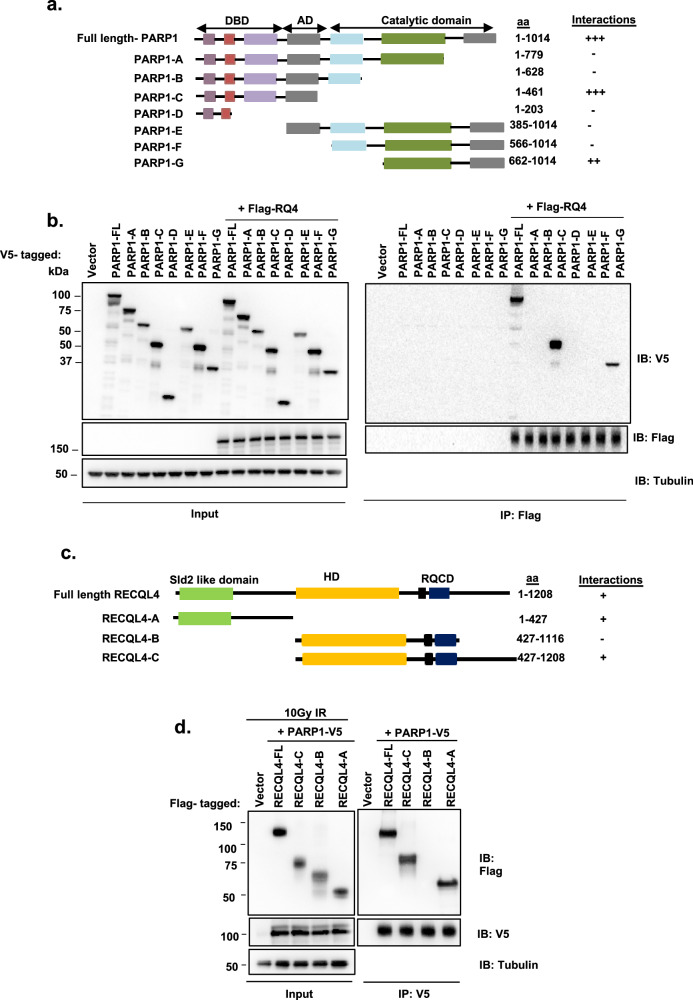
Identification of the PARP1 domains required for interaction with RECQL4. **a**, An illustration of V5-tagged full-length PARP1 and fragments used for binding assays. The numbers denote amino acid (aa) residues. The results from **b** are scored in the right column as RECQL4 binding. **b**, Cell extracts from HEK293T cells expressing V5-tagged full-length or fragments of PARP1 with or without Flag–RECQL4 were immunoprecipitated with an anti-FLAG antibody, followed by immuno blotting (IB) with an anti-V5 antibody. **c**, An illustration of Flag-tagged full-length RECQL4 and fragments used for binding assays. The numbers denote amino acid residues. Sld2-like domain and HD. The results from **d** are scored with + in the right column as PARP1 binding. **d**, After 10 Gy of IR DNA damage, extracts from HEK293T cells expressing Flag-tagged full-length or fragments of RECQL4 with V5-tagged PARP1 were immunoprecipitated with an anti-V5 antibody, followed by western blotting with anti-Flag and anti-V5 antibodies.

PARP1 is a multidomain protein containing an N-terminal DNA binding domain (DBD), an automodification domain (AD) with a breast cancer 1 protein (BRCA1) C-terminal motif and a C-terminal catalytic domain^21^. To identify the protein regions that mediate interactions between PARP1 and RECQL4, we obtained V5-tagged full-length PARP1 and several deletion mutants (Fig. 4a) and overexpressed them in HEK293T cells (Fig. 4b). These constructs were cotransfected with or without Flag-tagged RECQL4, followed by co-IP with anti-Flag beads and western blotting with an anti-V5 antibody. Only domains C (amino acids 1–461) and G (aa 662–1,014) of PARP1 (Fig. 4b) coimmunoprecipitated with RECQL4 with the same efficiency as full-length PARP1 (Fig. 4b). These results suggest the presence of multiple points of contact between PARP1 and RECQL4.

Similarly, RECQL4 is composed of several domains, including a Sld-2-like (SLD) domain, a basic region, a helicase domain (HD) and a RECQ conserved (RQC) domain (Fig. 4c). Consistent with a previous report that reported that the C-terminus of RECQL4 interacts with PARP1^49^, we found that both the N- and C-terminal regions of RECQL4 bind to PARP1 after DNA damage (Fig. 4c, d). We also confirmed these results using a PLA between Flag-tagged RECQL4 WT and its domains with PARP1 in U2OS WT cells (Supplementary Fig. 6). Notably, a majority of RECQL4-interacting protein partners interact with the SLD domain of RECQL4 (refs. ^50–52^). The combined regulation of these protein interactions remains to be determined, and some of them are regulated by phosphorylation and perhaps other post-translational modifications of RECQL4 (refs. ^12,50^).

### Role of RECQL4 in alt-NHEJ

**Fig. 5:**
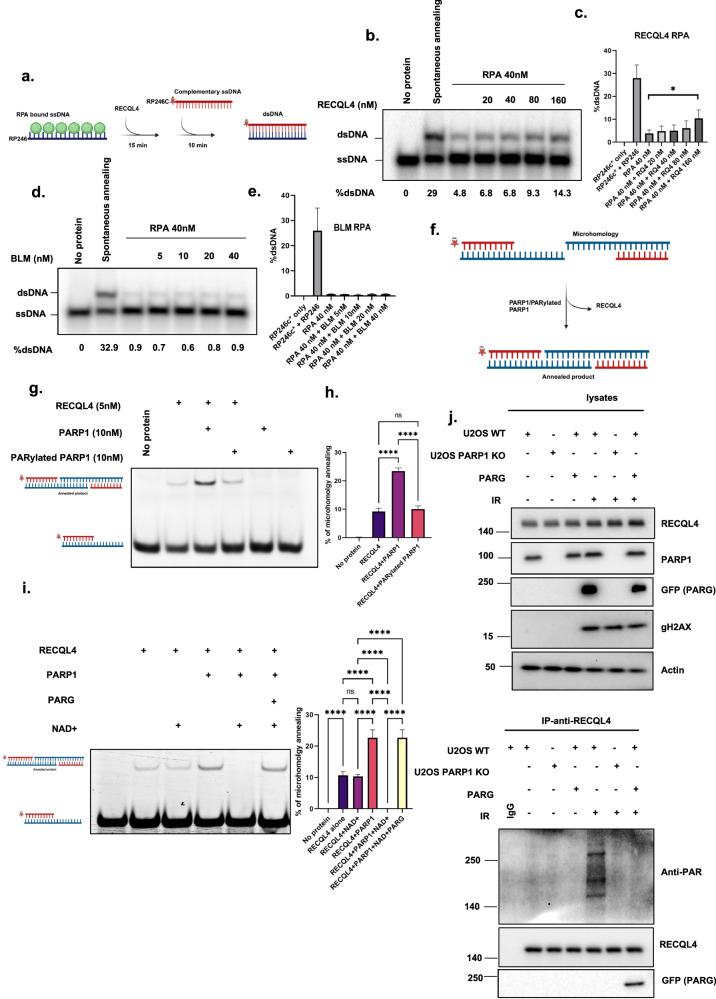
RECQL4 displaces RPA to promote microhomology-mediated annealing in alt-NHEJ. **a**, A schematic diagram of the annealing assay used to study whether RECQL4 stimulates the annealing of RPA-bound ssDNA. **b**, A representative nondenaturing gel showing ssDNA annealing in the presence of RPA with the indicated amounts of RECQL4. The percentage of dsDNA is indicated. **c**, A graph showing a quantitative representation of the data in **b**. **d**, Representative nondenaturing gel showing ssDNA annealing in the presence of RPA with the indicated amount of BLM. The percentage of dsDNA is indicated. **e**, Graph showing a quantitative representation of the data in **d**. **f**, A schematic diagram of the in vitro end-joining assay. **g**, A nondenaturing gel showing RECQL4-mediated microhomology annealing in the presence of the mentioned protein concentrations. **h**, A graph showing a quantitative representation of the data in **g**. **i**, A nondenaturing gel showing RECQL4 (5 nM)-mediated microhomology annealing in the presence of PARP1 (10 nM), with or without NAD^+^ (20 μM), and in the presence or absence of PARG (20 nM). The accompanying graph represents quantitative data derived from three independent experiments. **j**, GFP–PARG-expressing U2OS WT or PARP1 KO cells subjected to 10 Gy of gamma IR. Western blot analysis was performed for the indicated proteins. IP was conducted with an anti-RECQL4 antibody to assess PARylation with an anti-PAR antibody and to examine PARG interaction with an anti-GFP (PARG) antibody.

Our earlier findings clearly revealed that PARP1 selectively enhances the annealing activity of RECQL4 and that RECQL4 PARylation is essential for its recruitment to DSBs. Our next objective was to investigate the specific DNA repair pathway in which RECQL4 utilizes this annealing function. The alt-NHEJ pathway necessitates the microhomology annealing of DNA end products, providing a potential context for the annealing activity of RECQL4. Notably, RPA has been previously shown to be a negative regulator of alt-NHEJ^53^, and other RecQ helicases are known to physically and functionally interact with RPA. Recently, RECQL4 was shown to participate in classical-NHEJ (c-NHEJ)^54^. However, the role of RECQL4 in alt-NHEJ is unknown. Thus, we investigated the molecular mechanisms by which RECQL4 may promote alt-NHEJ. We hypothesized that RECQL4 may help displace RPA from ssDNA to facilitate the annealing of ssDNA microhomology overhangs, which is a crucial step in the alt-NHEJ pathway. To test this hypothesis, we performed an RPA displacement assay (Fig. 5a) using ssDNA, which was preincubated with RPA and then incubated with increasing amounts of RECQL4 and radiolabeled complementary ssDNA. The addition of RPA prevented spontaneous annealing of ssDNA, whereas the addition of RECQL4 stimulated modest ssDNA annealing to form double-strand DNA, indicating the weak displacement activity of RPA (Fig. 5b, c). To explore whether the limited annealing function of RECQL4 stems from its helicase activity, we utilized a helicase-inactive RECQL4 variant, K508A, in our experiments. The results, illustrated in Supplementary Fig. 7a revealed that both the WT RECQL4 and the helicase-inactive variant exhibited comparable activities. This finding aligns with existing studies indicating that the annealing capacity of RECQL4 is distinct from its helicase function^13^. In contrast, BLM did not result in increased ssDNA annealing in the presence of RPA (Fig. 5d, e). The percentage of dsDNA was calculated by measuring the number of dsDNA duplexes formed before and after the addition of RECQL4 or BLM. The protein ratio was assessed from initial standardization assays.

Alt-NHEJ uses short stretches of microhomology to facilitate DSBR^55^. To assess the involvement of RECQL4, we measured its microhomology annealing efficiency using model substrates with 10 base microhomology, as depicted in Fig. 5f. RECQL4 effectively annealed these substrates, forming double-sized products. Interestingly, only non-PARylated PARP1 stimulated RECQL4 microhomology annealing, whereas PARylated PARP1 did not differ from RECQL4 alone (Fig. 5g, h). Since PARG is a key dePARylation enzyme at DNA DSBR sites that regulate DNA repair proteins, we used PARG and NAD^+^ in our in vitro system to investigate how PARylation and dePARylation influence the annealing function of RECQL4. As shown in Fig. 5i, the annealing activity of RECQL4 was inhibited in the presence of NAD^+^ and PARP1. However, the addition of PARG restored the annealing activity of RECQL4 to a level comparable to that of unmodified RECQL4 and PARP1 together. These findings suggest that PARylation by PARP1 suppresses the annealing activity of RECQL4, whereas PARG removes this inhibition of RECQL4. Furthermore, through IP of RECQL4 we confirmed that PARG dePARylated RECQL4 following 10 Gy of IR DNA damage (Fig. 5j).

To elucidate how PARP1 expression or PARP1 activity affects the interaction of RECQL4 with alt-NHEJ proteins, we performed GFP–RECQL4 IP after PARP1 knockdown or PARPi treatment before and after IR. As shown in Supplementary Fig. 7b, after DNA damage, RECQL4 associated with the alt-NHEJ proteins XRCC1, PARP1 and LIG3 in WT cells^56,57^; however, in PARP1-depleted or PARPi-treated cells, the interaction of RECQL4 with the alt-NHEJ proteins was abrogated. Previously, we demonstrated that MRE11 regulates the retention of RECQL4 at DSBs^12^. To investigate whether MRE11 has any role in RECQL4 function in TMEJ, we conducted a similar experiment in the presence of Mirin, an inhibitor of MRE11 nuclease activity. As shown in Supplementary Fig. 7c, olaparib reduced the interaction of RECQL4 with Lig3 (a TMEJ protein), whereas this interaction was unaffected in Mirin-treated cells. In summary, RECQL4 removes RPA from ssDNA and promotes annealing of MMEJ. PARP1 expression and PARP1 activity are essential for the interaction of RECQL4 with alt-NHEJ repair proteins.

### RECQL4 is required for efficient DSBR

**Fig. 6:**
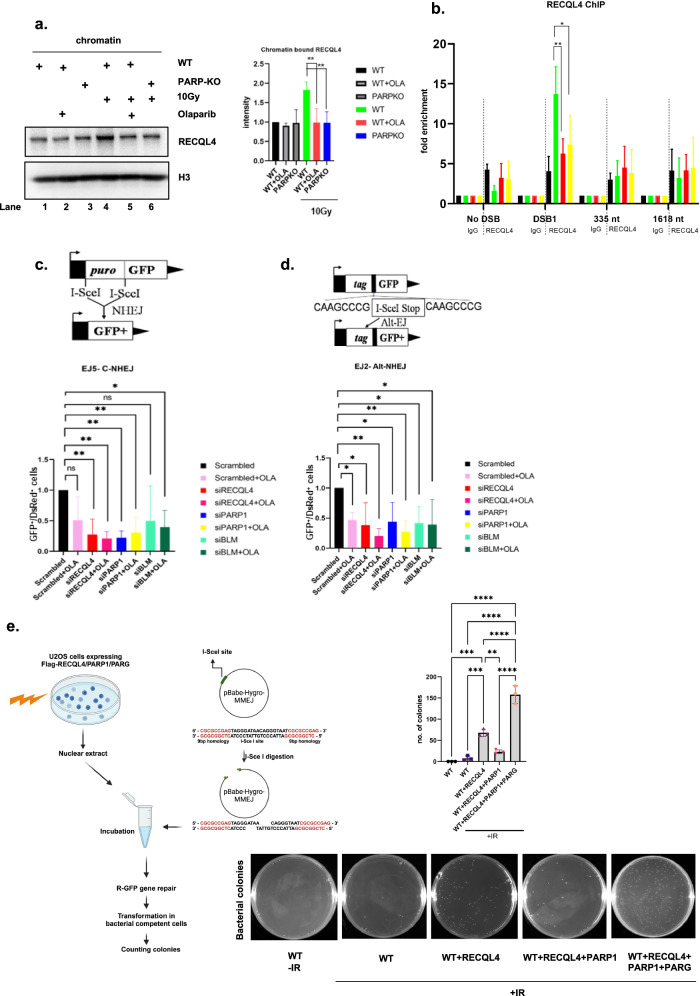
Loss of RECQL4 diminishes the DSBR pathways NHEJ and alt-NHEJ. **a**, The effect of PARP1 and its activity on the chromatin binding of RECQL4 after DNA damage. Chromatin fractions were prepared from U2OS WT/PARP1 KO cells before and after 10 Gy of IR DNA damage with or without 5 µM olaparib treatment. Western blot analysis was performed using an anti-RECQL4 antibody. Anti-H3 served as a chromatin marker and loading control. The graph shows the quantitative representation of chromatin-bound RECQL4. A Student’s *t*-test (two-sided) was performed to assess statistical significance (*P < 0.05, **P < 0.01 and ***P < 0.001). **b**, ChIP analysis of RECQL4 occupancy at AsiSi-induced DSBs. ChIP analysis was performed in AsiSi–ER–U2OS cells before (−) and after (+) 4 h of 4-OHT treatment and with or without PARP1 inhibition (Ola) or knockdown (siPARP) using rabbit IgG or anti-RECQL4 antibodies. Enrichment was assessed via real-time qPCR amplification using primers proximal and distal to the AsiSi-induced DSB site on a specific chromosome. The error bars represent the s.e.m. A two-way ANOVA was performed to assess significant differences (*P < 0.05, **P < 0.01 and ***P < 0.001). **c**, **d**, Schematic images of the results of the cellular GFP reporter cassette DNA repair assays, with the effects of RECQL4, BLM and PARP1 inhibitors on the efficiency of repairing I-SceI-mediated DSBs through the c-NHEJ (**c**) and alt-NHEJ (**d**) pathways. RECQL4 inhibits NHEJ with or without a PARP1 inhibitor (olaparib) 24 h post-siRNA transfection (**c**). EJ5 cells were cotransfected with plasmids expressing I-SceI and DsRed constructs, and the relative NHEJ efficiency was measured. RECQL4 inhibits alt-NHEJ in PARP1 inhibitor (olaparib)-treated cells (**d**). Plasmid-transfected cells were treated with olaparib for 4 days, after which NHEJ and alt-NHEJ efficiency were measured. The error bars represent the s.e.m. of three independent experiments. The repair efficiency of each repair pathway is reported relative to the siControl condition, which is set arbitrarily to 1.0. All experiments were repeated at least three times, and the error bars represent the s.e.m. A two-way ANOVA was performed to assess significant differences (*P < 0.05, **P < 0.01 and ***P < 0.001). **e**, A schematic outline of the MMEJ in vitro assay (left). Mean number ± s.d. of colonies obtained from the in vitro MMEJ assay using nuclear lysates from U2OS cells expressing Flag–RECQL4 alone or with V5-PARP1 in the presence or absence of GFP–PARG after 10 Gy IR from three independent experiments; ***P < 0.001 and ****P < 0.0001 using one-way ANOVA (right top). Representative images of colonies harboring the repaired pBABE-hygro-MMEJ plasmid (right bottom).

Our next aim was to investigate the impact of PARP1 on the chromatin binding affinity of RECQL4 following DNA damage. WT or PARP KO cells were treated with olaparib and/or exposed to 10 Gy of IR. Chromatin fractions were isolated as described in the Materials and methods. As depicted in Fig. 6a, in cells without IR treatment, a basal level of RECQL4 was bound to chromatin (lane 1). While RECQL4 participates in various DNA repair pathways, it also plays an important role in the initiation of DNA replication and interacts with replication origins. Notably, the SLD2-like domain of human RECQL4 forms a chromatin-bound protein complex during the cell cycle, involving core replication factors such as the MCM complex, CDC45 and GINS^58–60^. Therefore, chromatin-bound RECQL4 is present even in the absence of DNA damage. Furthermore, the association of RECQL4 with chromatin appeared to be largely independent of PARP1 or its activity, as demonstrated by minimal effects on RECQL4 retention upon PARP1 depletion or olaparib treatment (lanes 2 and 3). However, after IR, the purified chromatin fraction presented increased RECQL4 (lane 4). In contrast, lanes containing PARP1-knockdown cells or cells treated with PARPi exhibited significantly fewer chromatin-bound RECQL4 proteins (lanes 5 and 6) (Fig. 6a). These results suggest that RECQL4 utilizes at least two distinct modes for chromatin binding: one that is independent of PARP1 and its activity (basal binding) and another that is DNA damage- and PARP1-dependent.

PARP1 recognizes DNA lesions and is involved in the recruitment of multiple DDR proteins, which facilitates efficient DNA repair^25,61^. To better dissect how PARP1 affects RECQL4 distribution at DSB sites, we took advantage of the U2OS AsiSi DiVa system, where DSBs can be induced at specific AsiSi target sequences across the human genome^62,63^. After knockdown of PARP1 or PARPi treatment, U2OS AsiSi–ER cells were treated with 4-OHT for 4 h, after which RECQL4 ChIP using an RECQL4 antibody was performed. Induction of DSBs with 4-OHT consistently increased RECQL4 occupation at the DSB site (DSB1) in WT cells. Neither the PARP1-knockdown nor the PARPi-treated cells exhibited increased RECQL4 occupancy at DSB1 (Fig. 6b). Collectively, these data demonstrate that PARP1 regulates RECQL4 binding to chromatin after DNA damage and occupancy at DSB sites.

Human cells possess multiple different DSBR pathways, including HR, c-NHEJ, alt-NHEJ and single-strand annealing^17,42^. PARP1 is involved in the HR^34,64^ and alt-NHEJ pathways^65^, and RECQL4 is required for c-NHEJ-mediated^11^ and HR-mediated^12^ DSBR. In contrast, BLM plays a role primarily in HR-mediated DSBR^66^. DSBR pathway choice is different in different phases of the cell cycle, and we previously reported that RECQL4 is phosphorylated, which facilitates the modulation of DSB pathway choice^50^. PARP1 is known to function in alt-NHEJ^67^, and here we have shown that RECQL4 interacts with PARP1 and that PARP1 protein expression and activity are required for RECQL4 recruitment to sites of damage. Therefore, to investigate RECQL4 in the DSBR via c-NHEJ and alt-NHEJ and whether loss of the PARP1 protein or activity modulates this function, we used cell-based GFP repair assays^68^. For this purpose, a site-specific I-SceI endonuclease was overexpressed in cells carrying an I-SceI target cleavage site in the marker gene *GFP* (Fig. 6c, d)^42,43^. After I-SceI introduced a DSB in *GFP*, the extent of DSBR was monitored as the change in fluorescence output by flow cytometry (FACS). Two cell lines were used, EJ2-GFP and EJ5-GFP, with different combinations of RECQL4, BLM and PARP1 depletion, with or without olaparib (Supplementary Fig. 8a). EJ2 reports the DSBR via the alt-NHEJ, and EJ5 monitors the DSBR via the c-NHEJ. Consistent with previous results^43^, olaparib inhibited alt-NHEJ-mediated DSBR but not c-NHEJ-mediated DSBR (Fig. 6c, d). The number of GFP-positive cells decreased significantly in both cell lines depleted of RECQL4 (Fig. 6c, d). RECQL4 has been implicated in c-NHEJ-mediated DSBR through its direct interaction with the MRN complex and through the initiation of DNA end resection with CtBP (carboxy-terminal binding protein) interacting protein (CtIP)^50^. However, the function of RECQL4 in alt-NHEJ-mediated DSBR is unknown. Interestingly, in both the alt-NHEJ and c-NHEJ reporter cell lines, cells treated with RECQL4 siRNA, PARP1 siRNA or olaparib showed significantly lower DNA repair capacity than cells treated with control siRNA. These results suggest that both PARP1 and RECQL4 play important roles in the alt-NHEJ and c-NHEJ pathways (Fig. 6c, d). In contrast, siRNA targeting BLM did not impair c-NHEJ-mediated DSBR, but it did decrease alt-NHEJ DSBR (Fig. 6c, d). Knockdown of BLM with or without olaparib did not significantly change the results. This finding is consistent with our in vitro and DNA recruitment results showing that depletion of PARP1 or PARPi treatment did not alter BLM function or recruitment. Therefore, loss of PARP1 does not appreciably alter BLM’s function in these repair assays.

Since PARP1-mediated PARylation plays a crucial role in the recruitment of RECQL4 to DNA break sites, we used a linearized plasmid as a DNA damage substrate and used nuclear extracts expressing RECQL4 alongside either PARP1 alone or PARP1 with PARG as protein sources to examine the effect of RECQL4 PARylation and dePARylation on its MMEJ efficiency. Nuclear and whole-cell extracts were prepared after DNA damage. RECQL4 was coexpressed with PARP1 and with both PARP1 and PARG (Supplementary Fig. 8b). To test MMEJ, the pBabe-hygro-MMEJ plasmid was digested with the I-SceI restriction enzyme and incubated with these nuclear extracts. Successful repair of the circular plasmids led to colony formation, with the colonies representing MMEJ-repaired plasmids. As shown in Fig. 6e, the expression of RECQL4 alone increased the number of MMEJ colonies. However, when coexpressed with PARP1, the colony count decreased. Interestingly, the addition of PARG counteracted the inhibitory effect of PARP1, resulting in a further increase in MMEJ colony formation. These findings suggest that dePARylation is required for RECQL4 to efficiently facilitate the repair of MMEJ substrates.

Polymerase Theta (PolQ) is a critical alt-NHEJ factor in mammalian cells. PolQ inhibition suppresses alt-NHEJ at dysfunctional telomeres and hinders chromosomal translocations at nontelomeric regions^55^. Genetic inactivation or inhibition of PolQ leads to synthetic lethality in HR-deficient BRCA2 KO cells^17,69^. To understand the epistatic role of RECQL4 and PolQi, we conducted a clonogenic assay in DLD1 WT cells transfected with either siControl or siBRCA2 (Supplementary Fig. 8c). Depleting RECQL4 or treating cells with PolQi in BRCA2-reduced cells decreased cell survival to 50%, and combining these approaches further reduced survival to 20%. Additionally, the application of olaparib resulted in complete cell death, as shown in Supplementary Fig. 8c. This finding suggests a cooperative function between RECQL4 and PolQ within the same DNA repair pathway.

## Discussion

Efficient repair of DSBs requires accurate annealing of complementary ssDNA ends at or near the site of the break. Proteins that promote the annealing of complementary ssDNA are common, but the mechanism that facilitates the search for sequence homology and the annealing process itself are poorly understood. In vitro experiments have shown that both RECQL4 and BLM play roles in promoting strand annealing. Specifically, RECQL4 exhibits robust annealing activity within the RECQ helicase family^18^.

The PARP1 and RecQ helicases are some of the earliest responders to DNA damage. Previous studies have shown strong associations of RECQL1, WRN and RECQL4 with PARP1^49,70,71^. Woo et al. demonstrated that RECQL4 is covalently PARylated by PARP1^49^. However, the dynamic role of PARP1 and PARG in RECQL4 recruitment and activity has remained unclear. The present study demonstrated that PARP1-mediated PARylation of RECQL4 plays a key role in its recruitment to DSB sites (Fig. 2). Furthermore, PARG removed the suppressive PARylation marks on RECQL4, which helped RECQL4 perform its DNA annealing activity (Fig. 5i). Here, we show that RECQL4 is rapidly recruited to DSBs in a PARP1- and PARylation-dependent manner (Fig. 2). Since olaparib treatment decreased the PARP1‒RECQL4 interaction after DNA damage (Fig. 3b) and affected the recruitment of RECQL4 to DSBs, we hypothesize that RECQL4 recruitment to DSBs depends on both the interaction between PARP1 and its PARylation activity. It has been shown that RECQL4 is PARylated both in vitro and in vivo^49^. We mapped the domains of RECQL4 that bind to PARP1 (Fig. 4) and found that they are different from the domains of WRN that bind to PARP1^72^. The N-terminal region of WRN interacts with the autoactivation domain of PARP1, whereas RECQL4 interacts with the N-terminal and catalytic domains of PARP1. We also found that RECQL4 interacts with PARP1 through its N- and C-terminal domains. A previous study reported that the N-terminal region of RECQL4 interacts with PARP1 (ref. ^49^). However, only a C-terminal construct was used to assay the interaction of RECQL4 with PARP1 by T7 phage display and the N-terminal region was not included. In the present study, we used all three domains of RECQL4 and observed that PARP1 interacts with both the N- and C-terminal regions of RECQL4. Our results were confirmed by IP (Fig. 4d) and proximity ligation assays (Supplementary Fig. 6). Thus, we have confirmed and expanded upon previous work showing the functional consequences of the RECQL4‒PARP1 interaction. These observations may explain why PARP1 has different effects on RECQL4 than on the other RecQ proteins, WRN and RECQL5^39^) Furthermore, we also observed that the RECQL4 N- and C-terminal domains have potential PAR binding sites (Fig. 3e), which can be further characterized in future studies.

PARP1 differentially stimulates strand annealing via RECQL4, whereas it inhibits strand annealing via BLM. Additionally, PARP1 inhibits RECQL4 DNA helicase activity but has no effect on BLM DNA helicase activity (Fig. 1). BLM has much stronger helicase activity than RECQL4 does, which may account for the different results (Fig. 1). Our previous study demonstrated that PARP1 and PAR inhibited the strand annealing and helicase activities of RECQL5 and WRN helicase^39^. The results of the present study demonstrate a clear difference in the in vitro activities of RECQL4 and BLM. Notably, non-PARylated PARP1 specifically stimulates the strand annealing activity of RECQL4 (Fig. 1). PARP1 binding to damaged DNA stimulates ADP ribosylation at many sites, both within PARP1 itself and on other proteins^73^. Our results indicate that the ssDNA annealing activity of RECQL4 is specifically stimulated by non-PARylated PARP1 and not by PARylated PARP1. These findings suggest that PARylated PARP1 may play a role in the recruitment of RECQL4 to DNA damage sites but does not affect the function of RECQL4.

Alt-NHEJ involves the annealing of short homologous repeats, known as microhomology, that flank a DSB^42,74^. Since RECQL4 has strong annealing activity, which is stimulated by PARP1, we hypothesized that the PARylation status of RECQL4 may modulate its role in alt-NHEJ^75^. While the recruitment of RECQL4 to DSB sites is PARylation dependent, we observed that the PARylation of RECQL4 inhibited its DNA annealing activity. This could be due to the strong negative charge of PAR. However, PARG-mediated removal of the suppressive effects of PAR on RECQL4 reactivated its annealing activity, resulting in more MMEJ-annealed products. RPA is required for many cellular processes, such as replication, recombination and DNA repair, by stabilizing ssDNA intermediates^76^. In alt-NHEJ, the resected ssDNA flanks are covered by RPA to prevent nucleolytic cleavage. For efficient alt-NHEJ, it is important to remove bound RPA followed by annealing of microhomologous strands. PolQ has been shown to be the only protein that regulates this process during alt-NHEJ. In vitro studies have demonstrated that PolQ efficiently removes RPA from resected DSBs and facilitates subsequent joining by alt-NHEJ^55^. Our in vitro results indicate that RECQL4 possesses similar RPA removal activity to that of PolQ. Since RECQL4 has stronger strand annealing activity and weakly dissociates from RPA, it is likely that RECQL4 and PolQ may work together during cellular alt-NHEJ or complement each other for efficient alt-NHEJ repair. The data presented here support this idea, as depletion of RECQL4 significantly inhibits alt-NHEJ and NHEJ (Fig. 6c, d). In addition, compared with olaparib alone, RECQL4 knockdown and olaparib treatment significantly inhibited alt-NHEJ. Compared with the control, knockdown of BLM also inhibited the alt-NHEJ repair pathway, but to a lesser extent than RECQL4 knockdown did (Fig. 6c, d). These results are consistent with the hypothesis that RECQL4 and PARP1 cooperate and play essential roles in alt-NHEJ. Notably, cellular c- and alt-NHEJ assays may not be sensitive enough to distinguish the roles of RECQL4, PARP1 and BLM. However, all these proteins play important and complex roles in DNA repair mechanisms. We used these assays primarily to understand the function of RECQL4 in the alt-NHEJ repair pathway.

Recent studies have revealed that alt-NHEJ is a backup pathway when HR or c-NHEJ fail to repair DNA damage^77^. Interestingly, more than 50% of high-grade ovarian cancers and 40% of sporadic breast cancers exhibit HR deficiency^78,79^. In particular, hyperactivation of alt-NHEJ has been reported in multiple cancers^80^, indicating the upregulation of alt-NHEJ in many cancers. PARP1, an important regulator of alt-NHEJ, is recognized as a critical target for the treatment of ovarian and breast cancer with four clinically approved drugs (olaparib, rucaparib, niraparib and talazoparib)^81^. Interestingly, increased RECQL4 expression has been reported in some cases of sporadic osteosarcoma. Increasing evidence suggests that RECQL4 protects cancer cells from endogenous and exogenous DNA damage. Our results show that PARP1/RECQL4 and PolQ cooperate in the alt-NHEJ pathway. In cancer cells deficient in HR, both a PolQ inhibitor and RECQL4 knockdown effectively eliminated these HR-deficient cells (Supplementary Fig. 8c). This observation suggests potential collaboration between RECQL4 and PolQ within the alt-NHEJ pathway. Therefore, combination therapy with RECQL4 or PolQ inhibition could be a potential cancer therapy approach. Inhibition of PARP function, either by depleting PARP1 or via the use of a PARP inhibitor, causes alt-NHEJ defects in multiple assay systems^37,82,83^. While the precise role of PARP1 in alt-NHEJ remains unclear, one of its roles is to recruit PolQ to DSBs^82^. PolQ plays a conserved role in alt-NHEJ, probably through its capacity to extend the templates that are stabilized by annealed microhomology^82^. During this step, RECQL4- and PARP1-mediated strand annealing may further stabilize broken DSB ends.

DNA strand annealing activity plays a critical role in physiological DNA repair processes by facilitating the accurate alignment and pairing of homologous DNA strands, a fundamental step in the repair of DSBs and the resolution of replication forks. This mechanism is essential for maintaining genomic stability, preventing mutations, and ensuring the correct repair of DNA damage, which is vital for cell survival and preventing disease states such as cancer. Through the restoration of genetic information with high fidelity, strand annealing supports the integrity of genetic material across cellular generations.

**Fig. 7:**
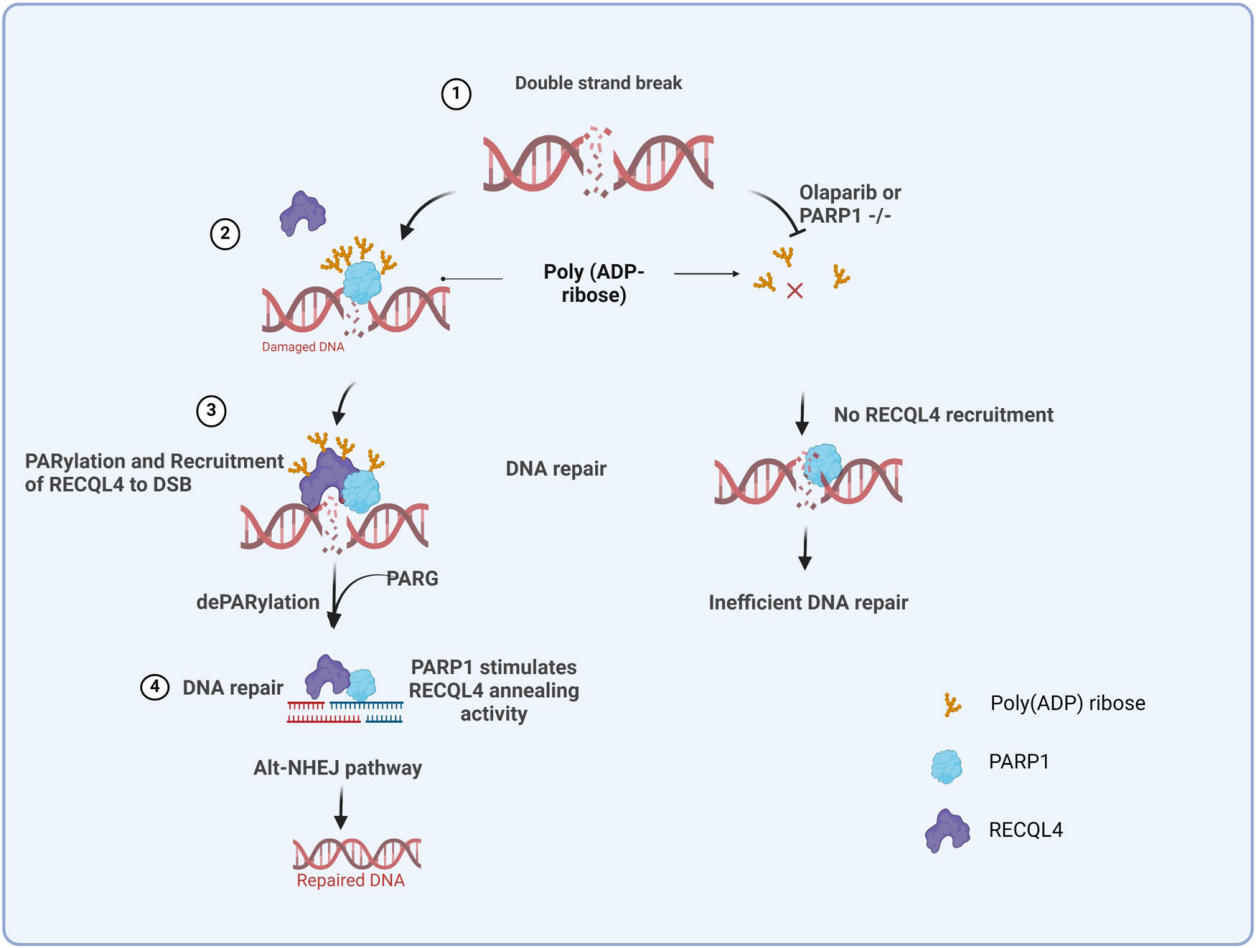
A model for the mechanism by which PARP1 regulates RECQL4 recruitment to DSB sites. After DNA damage, RECQL4 interacts with PARP1 and undergoes PARylation, which is essential for its recruitment at DSB sites to promote the alt-NHEJ pathway. PARP1 KO and PARPi treatment results in no PARylation of RECQL4 after DNA damage and hampers its recruitment to DNA damage sites. Furthermore, PARG removes PARylation repressive marks from PARP1 and RECQL4 so that they can perform their role in alt-NHEJ DNA repair. (The model was generated using BioRender).

On the basis of the above results and previous findings, we have constructed a model for how PARP1 and PARG regulate RECQL4 to promote DNA repair (Fig. 7). In this study, we demonstrate a two-step spatiotemporal mechanism within the alt-NHEJ pathway involving RECQL4 and PARP1. Initially, PARP1 PARylates RECQL4, which is required for the recruitment of RECQL4 to DSB sites. This PARylation of RECQL4 is a crucial step in the initial damage recognition and response phase. Following this, poly(ADP-ribose) glycohydrolase (PARG) removes PARylation marks from RECQL4 and PARP1. This dePARylation by PARG is essential, as it restores the DNA annealing activity of RECQL4, which is critical for the proper execution of the alt-NHEJ repair process. In summary, RECQL4 participates in multiple DSBR (c-NHEJ and alt-NHEJ) pathways, but it may cooperate with only PARP1 during alt-NHEJ. We show here how PARP1-mediated PARylation of RECQL4 facilitates its recruitment, while PARG removes the suppressive PAR modifications, reactivating the annealing activity of RECQL4. These findings may provide insight into the pathology associated with defects in or deficiency of human RECQL4 and its associated disorders.

## Supplementary information

Supplementary information
