# Supplementary material for: RECQL4 requires PARP1 for recruitment to DNA damage, and PARG dePARylation facilitates its associated role in end joining
Source: Exp Mol Med. 2025 Jan 28;57(1):264–80. doi: 10.1038/s12276-024-01383-z (PMC11799438; doi:10.1038/s12276-024-01383-z)
Supplement: Supplementary file 1 — Supplementary information [file 12276_2024_1383_MOESM1_ESM.pdf]

Supplementary Figures

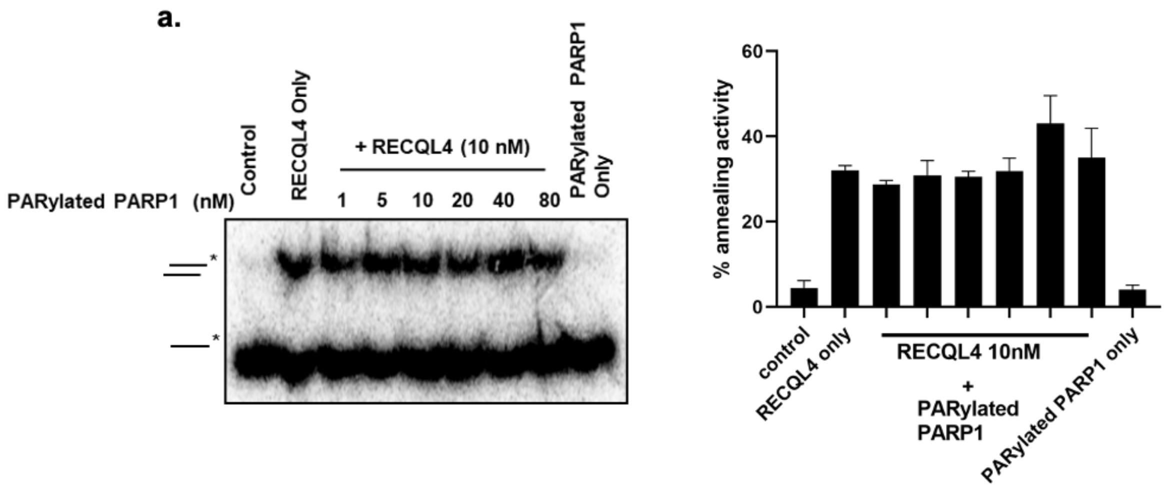

Supplementary Fig. 1: Strand annealing activity of RECQL4 and BLM in the presence of increasing concentration of PARylated PARP1. a. strand annealing activity of RECQL4 (10nM) was examined in the presence of increasing concentrations (0, 1, 5, 10, 20, 40, and 80 nM) of PARylated PARP1 with the radiolabeled ssDNA 80 mer DNA and its complementary single strand DNA.

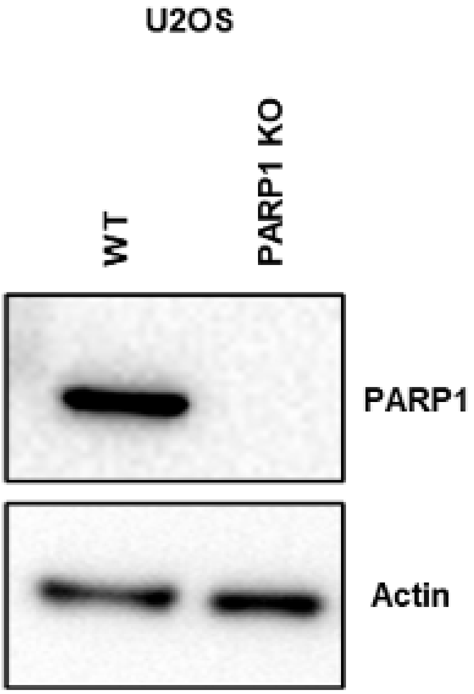

Supplementary Fig. 2: Western blotting for the wild type, PARP1 KO U2OS cell lines. The whole-cell extracts were immunoblotted with anti-PARP1 and anti-Actin antibodies. Actin serves as the endogenous loading control.

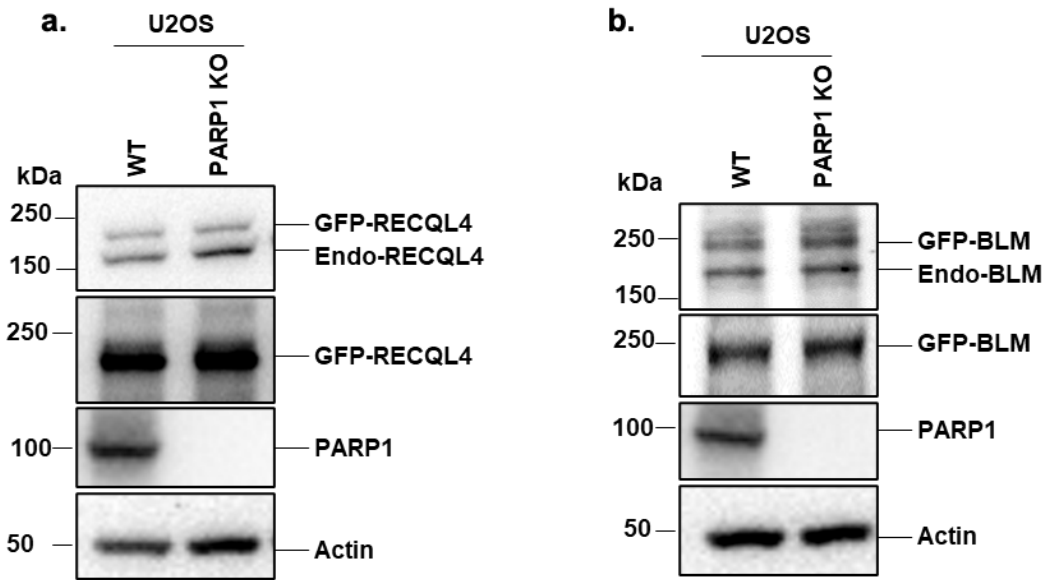

Supplementary Fig. 3: **western blotting showing expression of GFP-RECQL4/GFP-BLM**. GFP-RECQL4 (**a)** and GFP-BLM **(b)** expressed in U2OS WT/PARP1 KO cells. The whole cell extracts were probed using indicated antibodies. Actin served as loading control.

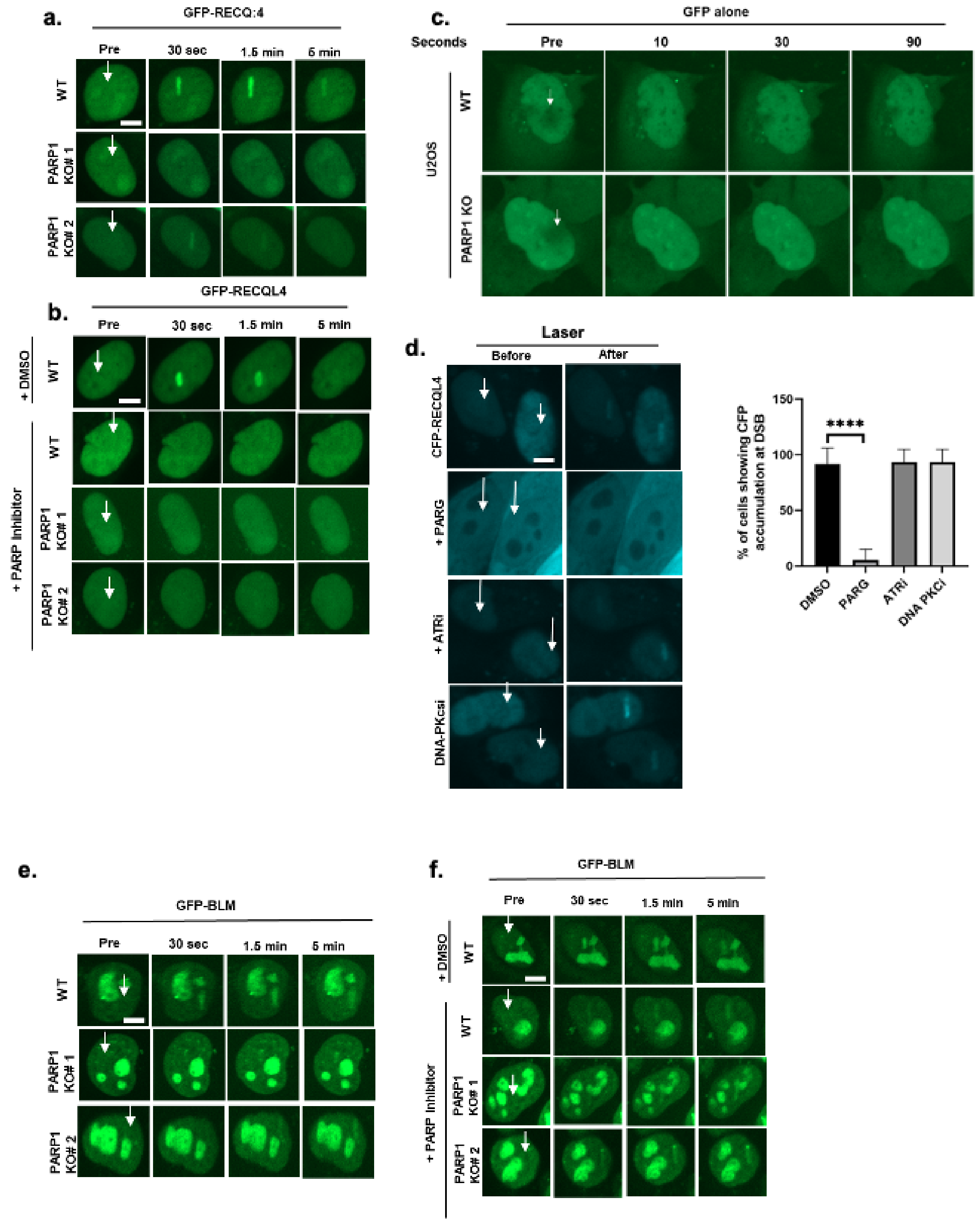

a.
GFP-RECQ:4
Pre
30 sec
1.5 min
5 min
WT
PARP1 KO# 1
PARP1 KO# 2
b.
GFP-RECQL4
Pre
30 sec
1.5 min
5 min
+ DMSO
WT
+ PARP inhibitor
WT
PARP1 KO# 1
PARP1 KO# 2
c.
GFP alone
Seconds
Pre
10
30
90
U2OS
WT
PARP1 KO
d.
Laser
Before
After
CFP-RECQL4
+ PARG
+ ATRi
DNA-PKcsi
% of cells showing CFP accumulation at DSB
150
100
50
0
****
DMSO
PARG
ATRi
DNA PKCi
e.
GFP-BLM
Pre
30 sec
1.5 min
5 min
WT
PARP1 KO# 1
PARP1 KO# 2
f.
GFP-BLM
Pre
30 sec
1.5 min
5 min
+ DMSO
WT
+ PARP inhibitor
WT
PARP1 KO# 1
PARP1 KO# 2

Supplementary Fig. 4: **PARP1-mediated PARylation is required for the early recruitment of RECQL4 to DSBs**. **a and b.** GFP-RECQL4 expressed in HeLa WT or PARP1 KO cells were pre-treated with or without 5 µM Olaparib for 3 h and then targeted with the 21% laser to induce DSBs. The white arrow indicates the laser striking area. **c.** GFP alone expressing U2OS WT or PARP1 KO cells are targeted with 21% laser to induce DSBs. The white arrow indicates the laser striking area. **d.** CFP-RECQL4 and PARG were overexpressed in U2OS WT cells. The relocation was monitored in a time course following laser micro-irradiation. Overexpressed CFP-RECQL4 U2OS WT cells were treated with ATR inhibitor or DNA-PKcs inhibitor or DMSO. The relocation kinetics of CFP-RECQL4 to DNA damage sites were examined and the graph was plotted as a percentage of cells showing recruitment of CFP-RECQL4 to DSBs. **e and f.** GFP-BLM expressed in HeLa WT or PARP1 KO cells were pre-treated with or without 5 µM Olaparib for 3 h and then targeted with the 21% laser to induce DSBs. The white arrow indicates the laser striking area.

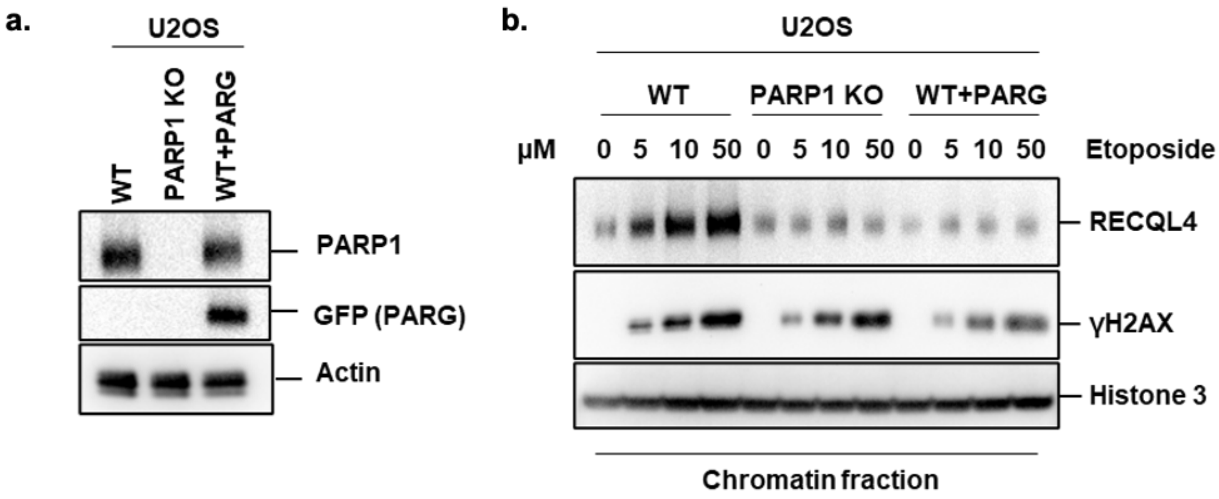

Supplementary Fig. 5: PARP1-mediated PARylation is essential for RECQL4 recruitment to DSBs in a dose-dependent manner after etoposide treatment. (a) Western blot showing expression of GFP-PARG in U2OS WT cells. **(b)** U2OS WT/PARP1 KO or GFP-PARG expressing cells treated with different concentrations of etoposide as indicated for 2 hours. The cells were collected, and chromatin fractionation was carried out for immunoblot using anti-RECQL4, anti-gH2AX, and anti-H3. Histone 3 serves as a loading control.

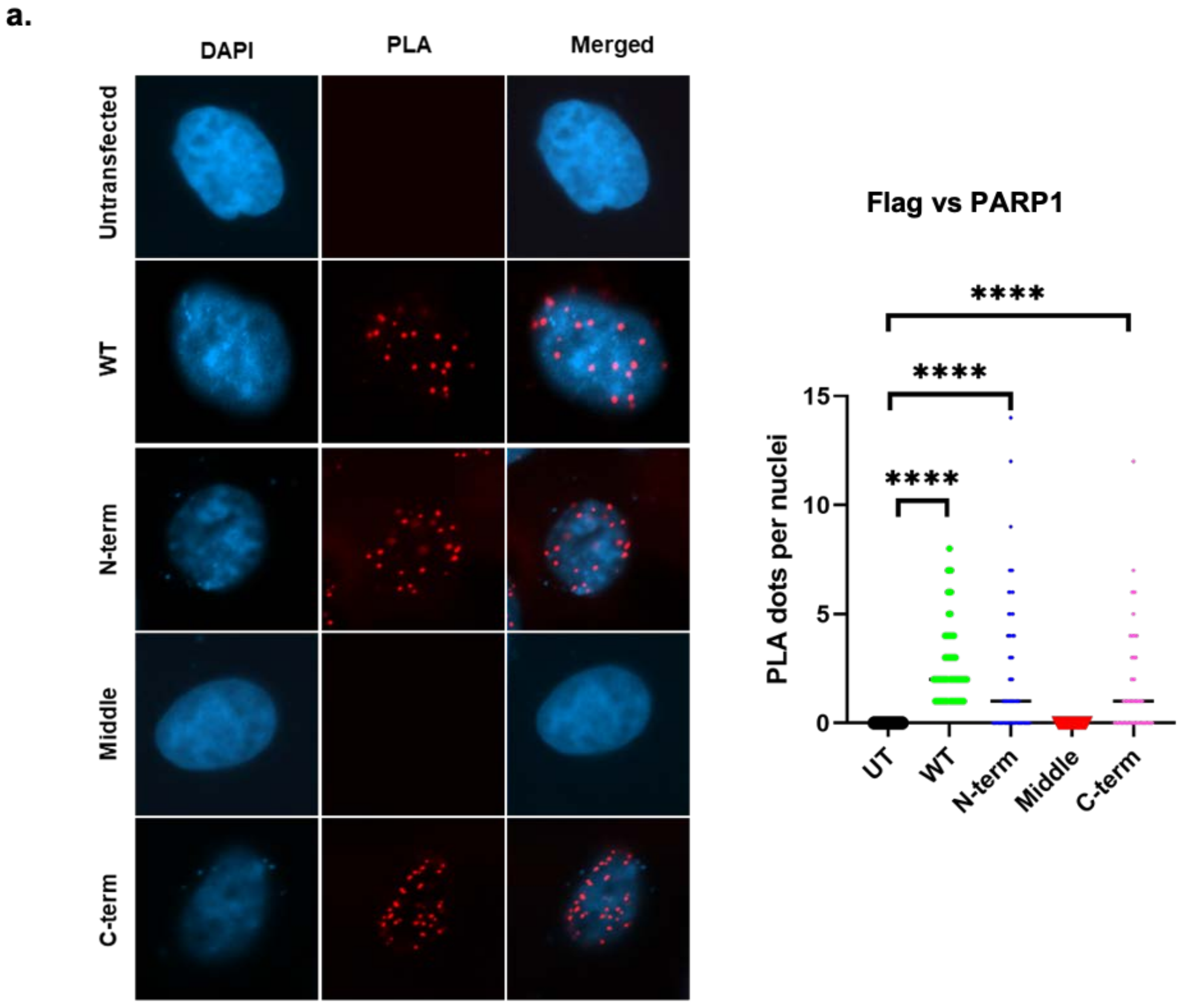

Supplementary Fig. 6: **PARP1 interacts with both RECQL4 N-ter and C-ter domains. a.** Proximity ligation assay (PLA) was carried out in cells expressing different regions of Flag tagged RECQL4. Cells were fixed using paraformaldehyde (PFA) and PLA was performed using anti-Flag (mouse) and anti-PARP1 (rabbit) antibodies. Images were taken under a microscope and PLA foci per nuclei were counted using cell profiler software. N=3, one way annova was performed to assess statistical significance (**** $P< 0.0001$).

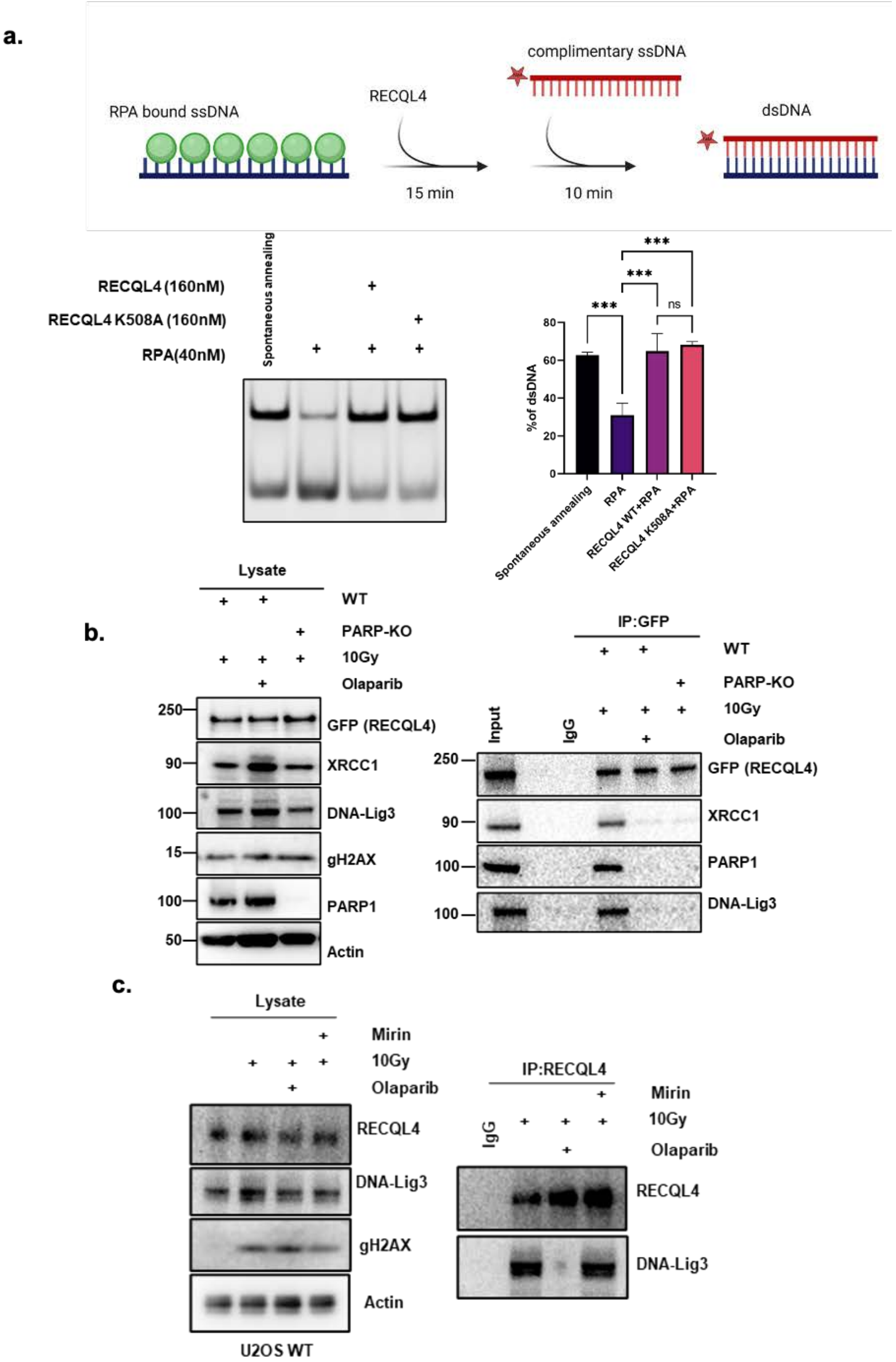
a.
RPA bound ssDNA
RECQL4
15 min
complimentary ssDNA
10 min
dsDNA
Spontaneous annealing
RECQL4 (160nM)
RECQL4 K508A (160nM)
RPA(40nM)
% of dsDNA
***
***
***
ns
Spontaneous annealing
RPA
RECQL4 WT+RPA
RECQL4 K508A+RPA
b.
Lysate
WT
PARP-KO
10Gy
Olaparib
GFP (RECQL4)
XRCC1
DNA-Lig3
gH2AX
PARP1
Actin
IP:GFP
Input
IgG
GFP (RECQL4)
XRCC1
PARP1
DNA-Lig3
c.
Lysate
Mirin
10Gy
Olaparib
RECQL4
DNA-Lig3
gH2AX
Actin
U2OS WT
IP:RECQL4
IgG
Mirin
10Gy
Olaparib
RECQL4
DNA-Lig3

Supplementary Fig. 7: RECQL4 anneals RPA-coated DNA substrates. a. Non-denaturing gel showing RECQL4 mediated annealing of RPA-coated DNA substrates in the presence of mentioned protein concentrations. The graph is the quantitative representation of the left panel. N=3, one-way annova was performed to assess statistical significance (**** $P < 0.0001$). **b.** Western blot analysis of indicated proteins immunoprecipitated along with RECQL4 from U2OS WT/PARP1 KO cells after 10Gy IR with or without 5uM olaparib treatment. All experiments were repeated at least three times. **c.** U2OS WT cells were either treated with Olaparib (5uM) or Mirin (30uM) one hour before 10Gy irradiation. After the DNA damage cell lysates were made and immunoprecipitation was carried using either anti-IgG (control) or anti-RECQL4 antibodies and probed using indicated antibodies.

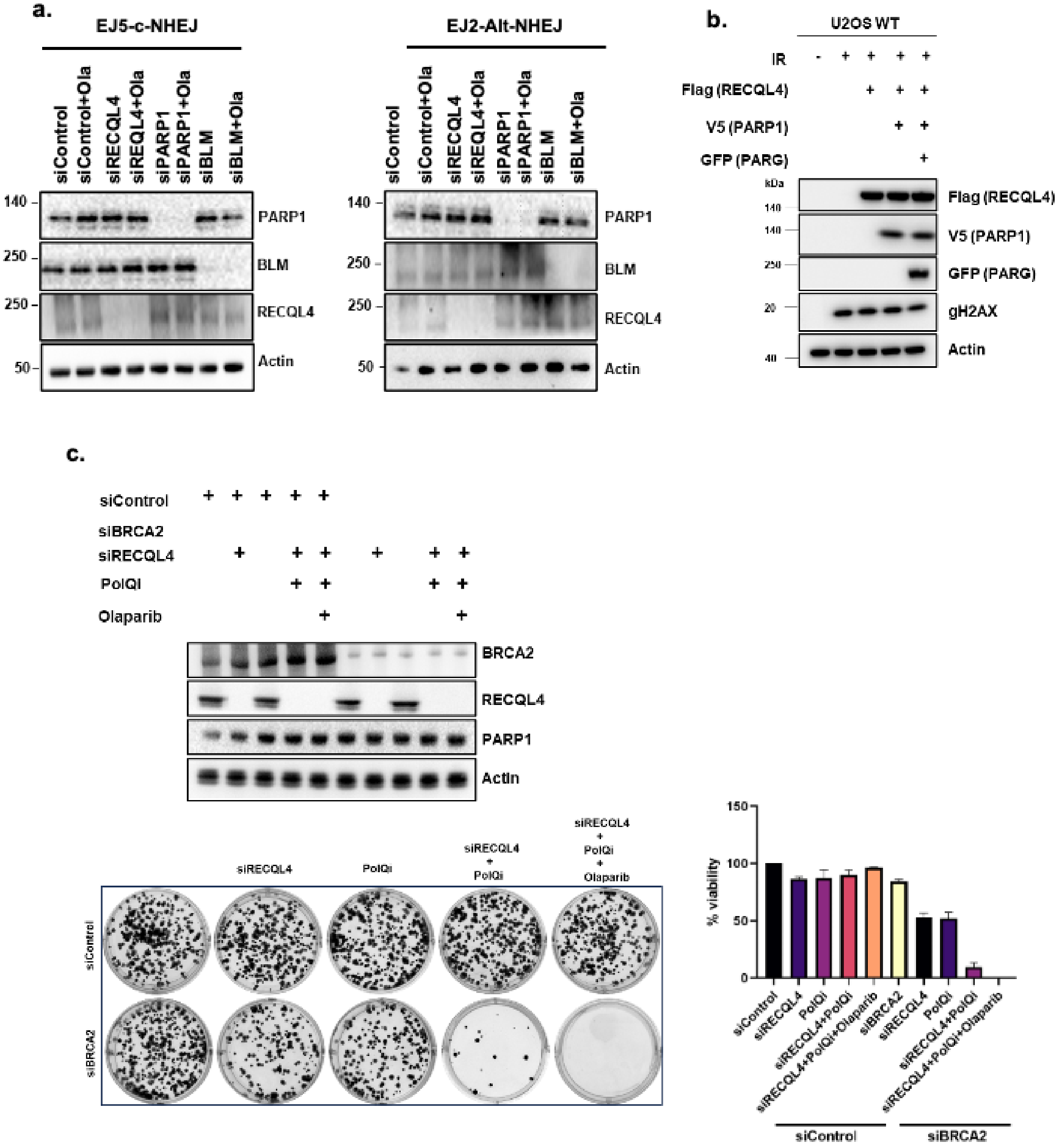
a.
EJ5-c-NHEJ
EJ2-Alt-NHEJ
siControl
siControl+Ola
siRECQL4
siRECQL4+Ola
siPARP1
siPARP1+Ola
siBLM
siBLM+Ola
140
250
50
PARP1
BLM
RECQL4
Actin
b.
U2OS WT
IR
Flag (RECQL4)
V5 (PARP1)
GFP (PARG)
kDa
140
250
20
40
gH2AX
c.
siBRCA2
PolQi
Olaparib
BRCA2
siRECQL4 + PolQi
siRECQL4 + PolQi + Olaparib
% viability
150
100
0
siRECQL4+PolQi
siRECQL4+PolQi+Olaparib

Supplementary Fig. 8: **a.** Western blot for the knockdown of PARP1, BLM, RECQL4 in U2OS EJ5 or EJ2 cells. **b.** Western blot analysis of exogenously expressed proteins in U2OS wild-type cells transfected with the specified plasmids. Cell lysates were collected 60 minutes after exposure to 10 Gy irradiation and probed with the corresponding antibodies. Actin was used as a loading control. **c.** RECQL4 knockdown and PolQi treatment increases synthetic lethality in HR deficient cells. DLD1 WT cells were transfected with either siControl or siBRCA2 with or without siRECQL4. After 72 hours post-transfection, 1000 cells from each group were plated in 6-well plates and treated with the specified inhibitors. The cells were allowed to form colonies over 14 days. Representative images of the colonies are provided. Bar graphs illustrate the clonogenic efficiency, calculated as the number of colonies formed divided by the total number of cells seeded, in siControl DLD1 WT. The data presented are the mean ± standard deviation (SD) from three independent experiments (n=3). BRCA2 and RECQL4 knockdown was confirmed through Western blotting, with actin served as the loading control.
